# High-Resolution Transcriptome Maps Reveal Strain-Specific Regulatory Features of Multiple *Campylobacter jejuni* Isolates

**DOI:** 10.1371/journal.pgen.1003495

**Published:** 2013-05-16

**Authors:** Gaurav Dugar, Alexander Herbig, Konrad U. Förstner, Nadja Heidrich, Richard Reinhardt, Kay Nieselt, Cynthia M. Sharma

**Affiliations:** 1 Research Center for Infectious Diseases (ZINF), University of Würzburg, Würzburg, Germany; 2 Integrative Transcriptomics, ZBIT (Center for Bioinformatics Tübingen), University of Tübingen, Tübingen, Germany; 3 Institute for Molecular Infection Biology, University of Würzburg, Würzburg, Germany; 4 Max Planck Genome Centre Cologne, Cologne, Germany; Uppsala University, Sweden

## Abstract

*Campylobacter jejuni* is currently the leading cause of bacterial gastroenteritis in humans. Comparison of multiple *Campylobacter* strains revealed a high genetic and phenotypic diversity. However, little is known about differences in transcriptome organization, gene expression, and small RNA (sRNA) repertoires. Here we present the first comparative primary transcriptome analysis based on the differential RNA–seq (dRNA–seq) of four *C. jejuni* isolates. Our approach includes a novel, generic method for the automated annotation of transcriptional start sites (TSS), which allowed us to provide genome-wide promoter maps in the analyzed strains. These global TSS maps are refined through the integration of a SuperGenome approach that allows for a comparative TSS annotation by mapping RNA–seq data of multiple strains into a common coordinate system derived from a whole-genome alignment. Considering the steadily increasing amount of RNA–seq studies, our automated TSS annotation will not only facilitate transcriptome annotation for a wider range of pro- and eukaryotes but can also be adapted for the analysis among different growth or stress conditions. Our comparative dRNA–seq analysis revealed conservation of most TSS, but also single-nucleotide-polymorphisms (SNP) in promoter regions, which lead to strain-specific transcriptional output. Furthermore, we identified strain-specific sRNA repertoires that could contribute to differential gene regulation among strains. In addition, we identified a novel minimal CRISPR-system in *Campylobacter* of the type-II CRISPR subtype, which relies on the host factor RNase III and a trans-encoded sRNA for maturation of crRNAs. This minimal system of *Campylobacter*, which seems active in only some strains, employs a unique maturation pathway, since the crRNAs are transcribed from individual promoters in the upstream repeats and thereby minimize the requirements for the maturation machinery. Overall, our study provides new insights into strain-specific transcriptome organization and sRNAs, and reveals genes that could modulate phenotypic variation among strains despite high conservation at the DNA level.

## Author Summary

Many species have evolved into diverse strains with phenotypic and genotypic variations that facilitate adaptation to different ecological niches and, in the case of pathogens, to different hosts. Whereas comparison of genome sequences reveals differences and similarities among strains, the consequences of genomic variations can be tracked by studying the functional output from the genome. RNA sequencing has been revolutionizing transcriptome analyses of both pro- and eukaryotes. However, the bioinformatics-based analysis is still lagging behind, and transcriptome features are often manually annotated, which is laborious and time-consuming. This is even more compounded for the analyses of multiple strains. Here we compared the primary transcriptomes of four isolates of *Campylobacter jejuni*, the leading cause of bacterial gastroenteritis in humans, and provide genome-wide transcriptional start site (TSS) maps using a novel automated annotation method. Our comparative RNA–seq showed that most TSS are conserved in multiple strains, but we also observed SNP–dependent promoter usage. Furthermore, we identified a novel minimal RNA–based CRISPR immune system as well as strain-specific small RNA repertoires. Our automated, comparative TSS annotation will facilitate and improve transcriptome annotation for a wider range of organisms and provides insights into the contribution of transcriptome differences to phenotypic variation among closely related species.

## Introduction

Deep RNA–sequencing (RNA–seq) has been revolutionizing transcriptome analyses of both pro- and eukaryotes [1], [2]. Several recent RNA–seq studies have revealed an unexpectedly complex transcriptional output from bacterial genomes and have been successfully used for the global identification of small RNA (sRNA) genes as well transcriptional start sites (TSS) [2], [3]. For example, our recently developed differential RNA–seq (dRNA–seq) approach, which is selective for the analysis of primary transcriptomes, allowed us to provide a genome-wide map of TSS in *Helicobacter pylori* [4]. In addition, a steadily growing number of studies report *cis-*encoded antisense RNAs as a widespread layer of gene-expression control in bacteria [5], [6]. Despite this rapid accumulation of transcriptome data, the bioinformatics-based data mining is still lagging behind. Thus, in most cases transcriptome features such as TSS and novel non-coding RNAs still have to be manually annotated, which is laborious and time-consuming. The problem is compounded for comparative transcriptomics of several species within a genus. Therefore, most RNA–seq studies have been limited to single bacterial strains so far (reviewed in [2], [3]). However, a comparative approach would not only allow for refining the transcriptome annotation of the individual species by integrating the information from multiple strains but can also reveal differences in transcriptome organization or gene expression among strains for which different phenotypes cannot be explained by the genome sequences alone.

Epsilonproteobacteria, including several important human pathogens such as *H. pylori* and *Campylobacter jejuni*, show a significant strain-to-strain variability on the phenotypic and genomic level and, thus, represent good model organisms for such a comparative approach. For example, multilocus sequence typing (MLST) analysis revealed that *C. jejuni*, the most prevalent food-borne bacterial pathogen in the industrialized world to date [7], [8], is genetically diverse, with a weakly clonal population structure and high rates of intraspecies recombination [9], [10]. It has been suggested that this extensive genetic diversity could lead to different clinical outcomes and facilitate adaptation to and persistence in the host [11], [12], [13]. Since comparison among multiple species or strains has mainly been examined at the genomic level, the differences in transcriptome structure that have an impact on phenotypic flexibility remain currently unknown [14], [15].

*C. jejuni* is a commensal of chicken but leads to gastroenteritis in humans, where it has also been associated with the development of secondary autoimmune disorders such as the Guillain-Barré or Miller-Fisher syndromes [16]. Except for a cytolethal distending toxin (CDT) [17] and homologs of a type-IV secretion system on the pVir plasmid of strain 81–176 [18], *C. jejuni* lacks most classical virulence factors of other gastrointestinal pathogens. Therefore, it has been suggested that mainly the motility and metabolic capabilities of *Campylobacter* are required for virulence and colonization of the host [16], [19].

Besides the lack of classical virulence determinants, little is known about the transcriptome structure of *Campylobacter*. The 1.6 megabase A/T-rich (∼31% G+C content) genome of *C. jejuni* encodes only three sigma factors and a few transcriptional regulators [20], indicating additional layers of gene regulation. Bacterial sRNAs are an emerging class of post-transcriptional gene expression regulators which have been implicated in bacterial stress response and virulence regulation [21]. As do 50% of all bacteria, *Campylobacter* lacks a homolog of the RNA chaperone Hfq, which is a key player in sRNA–mediated regulation in enterobacteria [22], [23]. Despite the prediction of five sRNA candidates in *C. jejuni* NCTC11168 using conventional, strand-insensitive RNA–seq combined with predictions of conserved RNA structures [24], knowledge about post-transcriptional regulation in this pathogen is still very limited. Furthermore, a global TSS map including sRNA and antisense RNA promoters is still missing. However, such sRNA regulators could also contribute to phenotypic diversity by mediating strain-specific gene regulation.

To gain insight into the sRNA repertoires as well as differences in primary transcriptomes among multiple strains of one bacterial species, we applied the dRNA–seq approach in a comparative manner to four *C. jejuni* isolates. In addition, we introduce a novel automated, comparative TSS annotation method for a fast and accurate generation of genome-wide TSS maps. Using this method we identified conserved and strain-specific TSS, some of which carry SNPs in promoter regions. Moreover, we detected 15 conserved and 24 strain-specific sRNA candidates, highlighting differential sRNA expression among strains. Furthermore, expression and conservation analysis reveals the presence of a minimal CRISPR system, an RNA–based immune system, in *C. jejuni*. Overall, defining the *Campylobacter* transcriptome structure including genome-wide promoter maps provides new insights into gene regulation and transcriptome evolution not only in this species but also in other pathogens.

## Results

### dRNA–seq analysis of multiple *Campylobacter jejuni* strains

The determination of exact transcript boundaries and identification of novel transcripts facilitates genome annotation and also the discovery of regulatory elements which control gene expression. Our recently developed dRNA–seq approach allows for an efficient global TSS annotation by differential sequencing of two cDNA libraries which discriminate primary and processed 5′ ends: one library (−) is generated from untreated total RNA, whereas the second library (+) is generated after treatment with terminator exonuclease (TEX) which specifically degrades processed RNAs with a 5′-mono-phosphate [4].

To identify conserved and strain-specific transcriptome features, we have applied the dRNA–seq approach to three human and one chicken isolate of *C. jejuni* (see Table 1 and Figure S1 in Text S1). Strain NCTC11168 [20], originally isolated from a case of human enteritis in the UK, was the first *Campylobacter* strain for which the genome was sequenced but displays only poor motility and virulence. *C. jejuni* 81–176 [25], isolated from a diarrheal outbreak in the U.S., is highly pathogenic and carries two large plasmids, pVir [18] and pTet [26]. Strain 81116 [27], a human isolate from a waterborne outbreak in the UK in 1983, is a genetically stable and lab-adapted strain which is still infective for chicken [28]. The virulence potential of the chicken isolate RM1221 [14] is unknown.

**Table 1:** Characteristics of *Campylobacter jejuni* strains used for dRNA–seq.

Feature	NCTC11168	81–176	81116	RM1221
**Source**	Human	Human	Human	Chicken
**Country**	UK	USA	UK	USA
**Serotype**	HS:2	HS:23,36	HS:6	HS:53
**Chromosome size [Mbp]**	1.64	1.62	1.63	1.78
**Number of ORFs** a	1,623	1,653	1,626	1,838
**G+C content [%]**	30.6	30.5	30.5	30.3
**Plasmids**	-	pVir, pTet	-	-
**Phage/genomic island elements**		6-kb integrated element (remnants of integrated plasmid)		CMLP1/
				CJIE1
				CJIE2
				CJIE3
				CJIE4
**CRISPR/Number of repeats**	yes/5	no	yes/8	yes/4
**Genome reference**	[20], [51]	[25]	[27]	[14]

a Number of ORFs according to NCBI annotations (May 2012).

Upon construction of two sets of dRNA–seq libraries from biological replicates of mid-log growth RNA samples for each of the four strains, we sequenced between 2.3 to 5.5 Mio cDNA reads per library which were subsequently mapped to the individual genome sequences (Table S1). Sequencing of dRNA–Seq libraries leads to a characteristic enrichment of cDNA reads at TSS in the TEX-treated sample [4]. These enrichment patterns allowed us to determine TSS in the four strains, and in many cases we observed an enrichment of cDNA reads at a given TSS in all four strains (for an example see Figure S2 in Text S1, *rpsL*). Moreover, our comparative dRNA–seq data showed that homologous genes might share the same TSS, even if the promoter regions are not conserved. Thus, based on sequence information alone it can be unclear whether a gene is expressed in the individual strains, whereas the comparative dRNA–seq data can provide this information. However, for several homologous genes, a clear TSS enrichment pattern (more than twofold in TEX+ *vs.* TEX−) was observed in only some of the strains despite highly conserved promoter regions (Figure S2 in Text S1, Cj1380). Nevertheless, a sharp flank in the cDNA distribution for all four strains indicates that transcription starts at exactly the same position. Without the comparative information from the other strains such cases could not unambiguously be defined as a TSS. Overall, a combination of sequence conservation and comparative TSS enrichment pattern analyses can be used to refine global TSS maps.

### Automated and comparative TSS analysis using a SuperGenome approach

**Figure 1:**
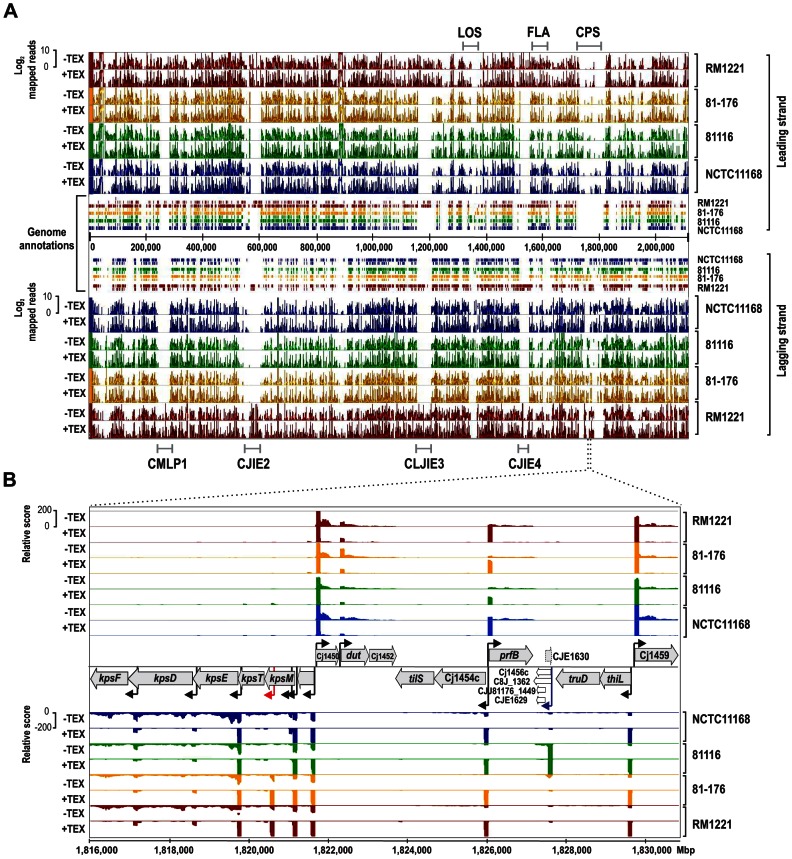
Differential RNA–seq and SuperGenome-based annotation of transcriptional start sites. (A) cDNA reads of −/+TEX libraries for the four *C. jejuni* strains were mapped to the SuperGenome, generated by whole genome alignment. All y-axes were set to same scale. Horizontal bars at the top and bottom indicate the highly variable LOS (lipooligosaccharide modification), FLA (flagellin modification), and CPS (capsule) gene loci as well as the integrated genome elements (CMLP1, CJIE2, CJIE3, and CJIE4) for strain RM1221. (B) Example region in the SuperGenome with mapped dRNA–seq reads of −/+TEX libraries of the four strains. TSS are indicated by black arrows. Red and blue arrows indicate an internal TSS within *kpsM*, which is missing in two of the strains, and a primary TSS which was only detected in strain 81116, respectively. All y-axes were set to same scale, which reflects a relative expression score. Note that gene CJE1630 (dotted arrow) is only annotated in RM1221 and that homologs of Cj1456c (white arrows) have different lengths in the four strains.

Manual annotation of TSS is labor- and time-consuming for a single strain and impractical for the analysis of multiple strains or larger genomes. To facilitate TSS comparisons, we mapped the four dRNA–seq data sets to a common coordinate system, the so-called SuperGenome [29], derived from a multiple whole genome alignment of the four strains (Figure 1A). Overall, 65% of the 2,115,275 SuperGenome positions were identical among the four strains (Table S2). The SuperGenome allows for a parallel visualization of the individual TEX+ and TEX- cDNA distributions of the four strains in a genome browser and thereby for a direct comparison of TSS enrichment patterns. The comparative dRNA–seq data show that the majority of TSS are enriched and detected in multiple strains (for an example locus see Figure 1B, black arrows) but that there are also differences among strains. For example, we observed a TSS within *kpsM*, which encodes for one of the capsule export genes, in only two of the strains and a TSS upstream of homologs of Cj1456c only in strain 81116 (Figure 1B, red arrow and blue arrows, respectively), indicating that there is strain-specific transcriptional output.

**Figure 2:**
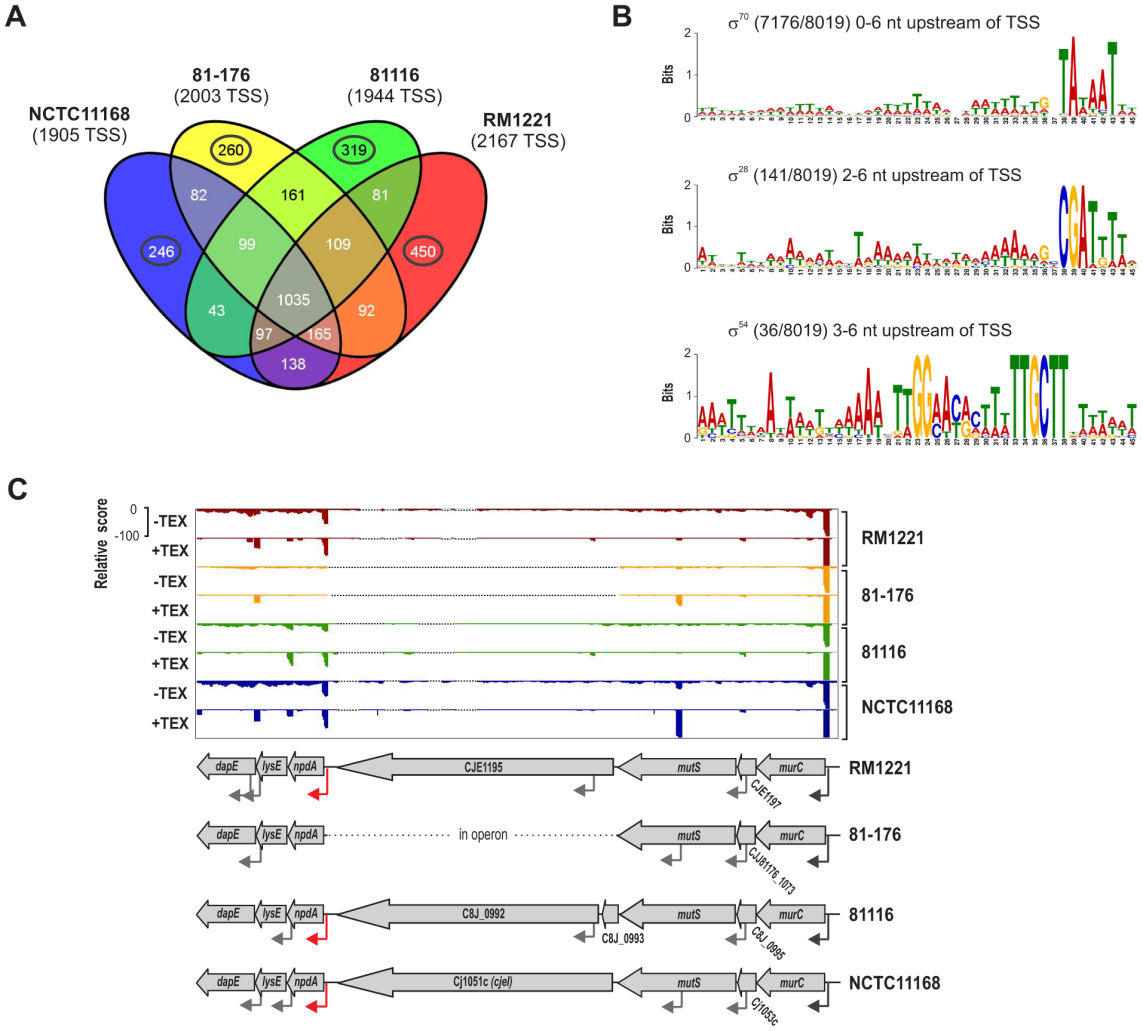
Transcriptome features of multiple *C. jejuni* strains. (A) Venn diagram showing the overlap among detected TSS between the four *C. jejuni* strains. The total numbers of TSS that were detected in each strain are indicated in brackets. TSS detected in single strains are marked by circles. See also Table 2. (B) Motif searches in the −50 to +1 sequences upstream of the 8,019 detected TSS in all four *C. jejuni* strains reveal promoter consensus sequence motifs for the three sigma factors, σ^70^ (RpoD), σ^28^ (FliA), and σ^54^ (RpoN). The numbers of sequences which contain the motif are indicated as well as the distances to the associated TSS. (C) dRNA–seq reads mapped to the SuperGenome between the *murC* and *dapE* genes in the four strains. In three strains, the *cjeI* gene is inserted together with a TSS (red arrow) for transcription of the downstream genes.

To automatically annotate TSS in a comparative manner, a two-step algorithm was employed: 1) TSS were detected independently for each strain based on dRNA–seq enrichment patterns and 2) TSS were mapped to the SuperGenome to allow for comparison and assignment of TSS among strains. Subsequently, all TSS of the individual strains were automatically classified as primary TSS (pTSS; main promoter of a gene) or secondary TSS (sTSS; alternative promoter upstream of pTSS of a gene), internal TSS (iTSS; promoter within gene), antisense TSS (asTSS; promoter antisense to a gene +/−100 nts) or orphan TSS (no association with annotation) according to their location relative to annotated genes (Figure S3A in Text S1 and Materials and Methods section). Our comparative approach enabled the annotation of a total of 3,377 TSS positions in the SuperGenome. 1,035 of the TSS were detected (but not necessarily enriched) in all four strains (Figure 2A, Table 2, and Table S3). Between 1,905 and 2,167 TSS were detected in the individual strains, including ∼300 strain-specific TSS (Table S4). The overall higher number of 2,167 TSS and 450 strain-specific TSS in RM1221 are mainly derived from TSS within its prophage elements (CMLP1, CJIE2-4) to which only cDNA reads from RM1221 were mapped in the SuperGenome (Table 1 and Figure 1A). On the two large plasmids, pVir (∼35 kB) and pTet (∼45 kB), of *C. jejuni* 81–176 we detected 70 and 58 TSS, respectively (Table S5). Approximately 60% of the conserved TSS are classified as pTSS or sTSS and in most cases drive transcription of mRNA genes. In contrast, only 21–31% of the strain-specific TSS are pTSS or sTSS and the majority of the strain-specific TSS (47–54%) are classified as antisense TSS (Table 2).

**Table 2:** Comparative TSS annotation.

	All TSS	pTSS	sTSS	iTSS	asTSS	Orphan
**All TSS in SuperGenome**	3377	973 (29%)	327 (10%)	1217 (36%)	1624 (48%)	61 (2%)
**TSS in individual strains**						
RM1221	2167	744 (34%)	202 (9%)	735 (34%)	988 (46%)	25 (1%)
NCTC11168	1905	675 (35%)	180 (9%)	653 (34%)	843 (44%)	17 (1%)
81–176	2003	676 (34%)	193 (10%)	651 (33%)	937 (47%)	20 (1%)
81116	1944	690 (35%)	181 (9%)	653 (34%)	856 (44%)	25 (1%)
**Conserved TSS**						
Detected in all strains	1035	527 (51%)	92 (9%)	328 (32%)	445 (43%)	10 (1%)
Detected in 2 or 3 strains	1067	204 (19%)	118 (11%)	398 (37%)	534 (50%)	36 (3%)
**Strain-specific TSS**						
RM1221	450	104 (23%)	39 (9%)	159 (35%)	235 (52%)	3 (1%)
NCTC11168	246	46 (19%)	19 (8%)	106 (43%)	119 (48%)	3 (1%)
81–176	260	34 (13%)	24 (9%)	98 (38%)	141 (54%)	4 (2%)
81116	319	58 (18%)	35 (11%)	128 (40%)	150 (47%)	5 (2%)
TSS missing in RM1221	99	21 (21%)	10 (10%)	35 (35%)	50 (51%)	3 (3%)
TSS missing in NCTC11168	109	22 (20%)	11 (10%)	36 (33%)	59 (54%)	4 (4%)
TSS missing in 81–176	97	25 (26%)	8 (8%)	38 (39%)	40 (41%)	4 (4%)
TSS missing in 81116	165	33 (20%)	24 (15%)	61 (37%)	83 (50%)	6 (4%)

This table lists the total numbers and fractions of the individual TSS classes for all SuperGenome TSS as well as conserved and strain-specific TSS detected in the four *C. jejuni* strains. pTSS: primary TSS, sTSS: secondary TSS, iTSS: internal TSS, asTSS: antisense TSS.

### Comparative analysis of regulatory elements

Using *MEME* [30] we analyzed the promoter regions (−50 to +1 of all TSS) upstream of the total number of 8,019 TSS in all four strains for sequence motifs of at least 45 nt. This revealed a periodic A/T-rich pattern instead of a clear −35 box followed by an extended −10 box (*TGxTATAAT*) for ∼89% of the promoter regions in the four strains as a consensus motif for the housekeeping sigma factor, σ^70^ (Figure 2B). This motif fits with a previously predicted consensus sequence for a smaller number of *Campylobacter* promoters and for σ^70^ in *H. pylori* based on dRNA–seq, indicating that transcription predominantly initiates at an extended −10 box in Epsilonproteobacteria [4], [31]. Moreover, we identified motifs which resemble the consensus binding sites for the alternative sigma factors, σ^28^ (FliA) and σ^54^ (RpoN), for 141/8,019 (1.8%) and 36/8,019 (0.4%) TSS, respectively (Figure 2B). Separate analyses of the four strains indicated that there is no strong variation in their general promoter patterns among strains (Figure S4 in Text S1). Since some genes that are known to be regulated by the alternative sigma factors were missed in our *MEME* searches, we defined a consensus motif based on nine and eight TSS from strain NCTC11168 with a previously described FliA- and RpoN-dependent promoter, respectively (Table S6). Pattern searches with this consensus motif revealed additional 138 and 26 TSS fitting the FliA and RpoN motifs, respectively (Table S7).

Approximately 35% of all TSS in each strain are pTSS and around 10% are classified as sTSS (Table 2 and Figure S3 in Text S1). The majority of the 3,241 5′UTRs defined by pTSS and sTSS of mRNA genes have a length between 20 and 50 nt (Figure S5A in Text S1). *MEME* searches detected an “AAGGA”-motif with an upstream A/T-rich sequence as a consensus for the ribosome binding site in 2,990 out of the 3,158 5′UTRs with a length ≥8 nt.

Pairwise comparison of the 5′UTR lengths of genes with at least one ortholog in one of the other strains showed that the majority of the conserved genes have the same 5′UTR length but revealed also several length variations among strains (Figure S5B in Text S1). Despite a correction - where possible - for differences in start codon annotations (Tables S8 and S9), many of the 5′UTR length differences are due to different 5′ ends of the CDS of the respective genes (Table S10). In some cases, different 5′UTR lengths result from insertion of new genes upstream of the start codon and the acquisition of new promoters in certain strains (Figure 2C). For example, in three of the strains, a gene encoding for a restriction modification (RM) enzyme (CJE1195, C8J_0992, *cjeI*) is inserted upstream of *npdA*, encoding a NAD-dependent deacetylase, which is transcribed from a pTSS that was probably acquired together with *cjeI*. In contrast, strain 81–176 lacks the RM enzyme as well as the downstream promoter and *npdA* is co-transcribed as a polycistronic mRNA from a TSS upstream of *murC*.

Besides insertions of new upstream genes with a novel promoter, different promoters can lead to 5′UTR length variation among strains. For example, different TSS sets were detected in the four strains for homologs of a putative transporter, Cj0339 (Figure S6A in Text S1). Moreover, it has previously been reported that the *asnA* gene of strain 81–176 has acquired a sec-dependent secretion signal to the otherwise cytoplasmic asparaginase found in NCTC11168 and facilitates asparagine utilization in this strain [19]. Our dRNA–seq data indicate that the different *asnA* forms are transcribed from strain-specific promoters with ∼100-fold higher cDNA read counts for the secreted asparaginase compared to the cytoplasmic form (Figure S6B in Text S1).

### SNP–dependent strain-specific promoter usage

**Figure 3:**
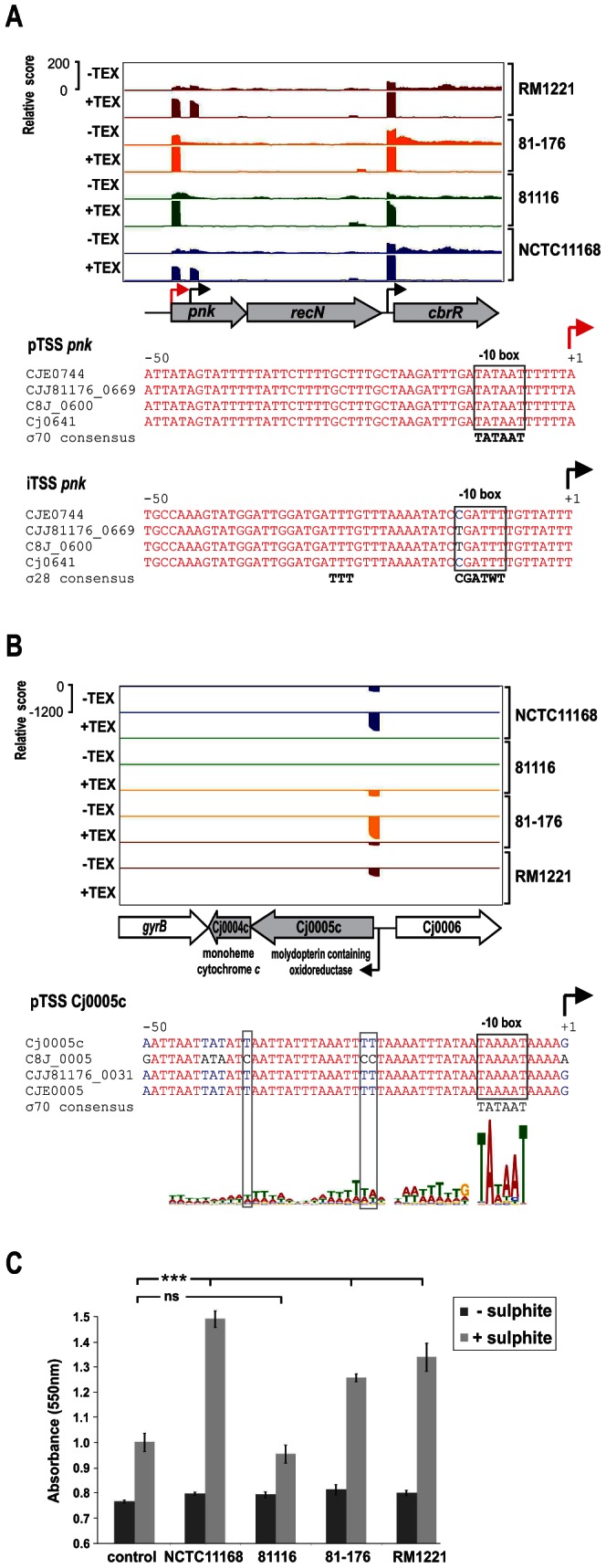
SNP–dependent promoter usage in *C. jejuni*. (A) dRNA–seq reads mapped to the *pnk-recN* operon in the SuperGenome. Red and black arrows indicate a primary (pTSS) and an internal TSS (iTSS), respectively. Corresponding alignments of the promoter region of the two TSS for *pnk* are shown. The iTSS within *pnk* is not expressed in two of the strains (81–176 and 81116) which carry a T to C exchange at the conserved “T” residue of the −10 box. (B) dRNA–seq reads mapped to the Cj0005c-Cj0004c operon, which encodes for a molydopterin containing oxidoreductase and monoheme cytochrome *c*. Promoter alignment of the primary TSS of Cj0005c shows disruptions in the A/T rich cyclic pattern upstream of the conserved σ^70^ −10 box shows in 81116. (C) Intact cells from the four *C. jejuni* strains were assayed for cytochrome *c* oxidoreductase activity in the absence (dark grey bars) and presence (light grey bars) of sulphite. The reduction of cytochrome *c* was measured as the increase in the absorbance at 550 nm. Strains NCTC11168, 81–176, and RM1221 showed a significant increase (p<0.0001) over basal levels (control without cells) of cytochrome *c* reduction when the samples were supplemented with sulphite.

Comparison of promoter motifs for conserved and strain-specific TSS revealed no difference in the general promoter patterns (Figure S7 in Text S1). However, our comparative TSS detection allowed us to identify regions with strain-specific promoter usage, e.g., regions for which we detected a TSS in only some of the strains although the region is present in the SuperGenome in all strains (Table S3, compare columns “mapCount” and “detCount”). We observed that in many cases single nucleotide polymorphisms (SNP) lead to disruption of promoters in a subset of strains (Figure 3). For example, the pTSS of *pnk*, encoding an inorganic polyphosphate/ATP-NAD kinase, is enriched and conserved in all four strains (Figure 3A). In contrast an iTSS within *pnk* is only detected in RM1221 and NCTC11168. In strains 81–176 and 81116 a C to T exchange in the “CGATTT” σ^28^ consensus seems to be sufficient to abolish transcription initiation from this TSS. Additional examples for mutations within conserved promoter elements are shown in Figure S8 in Text S1. Moreover, we noted that mutations within the periodic A/T-rich pattern upstream of the −10 box could also affect transcription (Figure 3B and Figure S9 in Text S1). For example, in strain NCTC11168, the Cj0004c and Cj0005c genes encode a monoheme cytochrome *c* and molybdopterin oxidoreductase, respectively, which allows *C. jejuni* to use sulphite as a respiratory electron donor [32]. This bi-cistronic operon along with the σ^70^ −10 box is conserved in all four strains (Figure 3B). However, mutations in the A/T rich upstream pattern of its pTSS in strain 81116 coincide with a loss of transcription either due to disturbing transcription initiation or binding of some regulatory factor. In line with this, we observed cytochrome *c* reduction as a measure for sulphite oxidation in only three of the strains (Figure 3C). This indicates, that although genes are conserved among strains and show high conservation in promoter regions, they may not necessarily be transcribed in all of them.

### The non-coding RNA repertoire of *C. jejuni*

**Figure 4:**
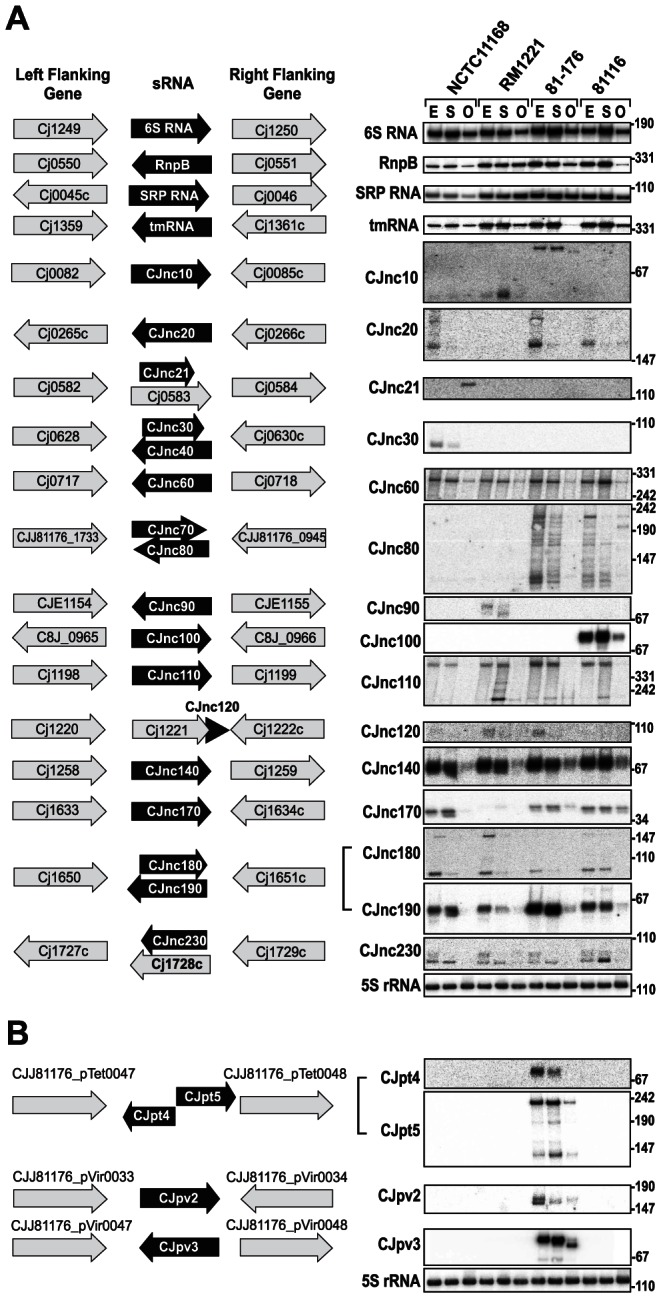
Small RNAs in *Campylobacter jejuni*. (A) *(Left)* Left and right flanking genes and orientation of sRNA candidates in *C. jejuni* NCTC11168. Arrows indicating the genes are not drawn to scale. Small RNA candidates were termed “CJncXXX” and numbered in steps of ten according to their genome position. *(Right)* Expression analysis of the housekeeping and candidate sRNAs during different growth phases in the four *C. jejuni* strains. Specifically, total RNA (15 µg per lane) was extracted at mid-exponential (E), stationary (S), and overnight (O) growth phase and analyzed by Northern blot using labeled DNA probes complementary to the sRNAs (see Table S14). The blots were probed for the housekeeping 5S rRNA as loading control. Note that the oligonucleotide probe for CJnc170 does not detect the homolog in strain RM1221 due to point mutations. (B) Genomic locations and Northern blots for sRNA candidates in the pVir and pTet plasmids of *C. jejuni* strain 81–176.

In addition to mRNA TSS, we identified many candidates for non-coding RNAs by our comparative dRNA–seq. For example, ∼45% of the TSS for each strain were classified as antisense TSS (asTSS) and 445 of the asTSS were detected in all four strains, indicating a large fraction of antisense transcription (Table 2, Figures S3 and S10 in Text S1). Furthermore, we detected several candidates for *trans*-encoded sRNAs in the chromosomes of the four strains and on the pVir and pTet plasmids (Figure 4, Figure S11 in Text S1 and Tables S11 and S12). Northern blot profiling under different growth phases confirmed expression of most of these sRNA candidates. Some sRNAs, such as CJnc60 or CJnc140, are highly conserved and show similar expression patterns in all strains. In contrast, some of the other conserved sRNAs, such as CJnc180 and CJnc190, which are encoded antisense to each other, show differential expression patterns among strains. Moreover, our Northern blots confirmed strain-specific sRNAs, such as CJnc30, which is only present in NCTC11168, or CJnc20, which is missing in RM1221. Most of our candidate sRNAs are transcribed from their own TSS, but we also found examples of sRNA candidates generated by processing, e.g., from 3′ ends of mRNAs (Figure S11 in Text S1 and Table S11). Furthermore, the majority of our sRNA candidates accumulated in exponential phase or during stationary phase growth.

**Figure 5:**
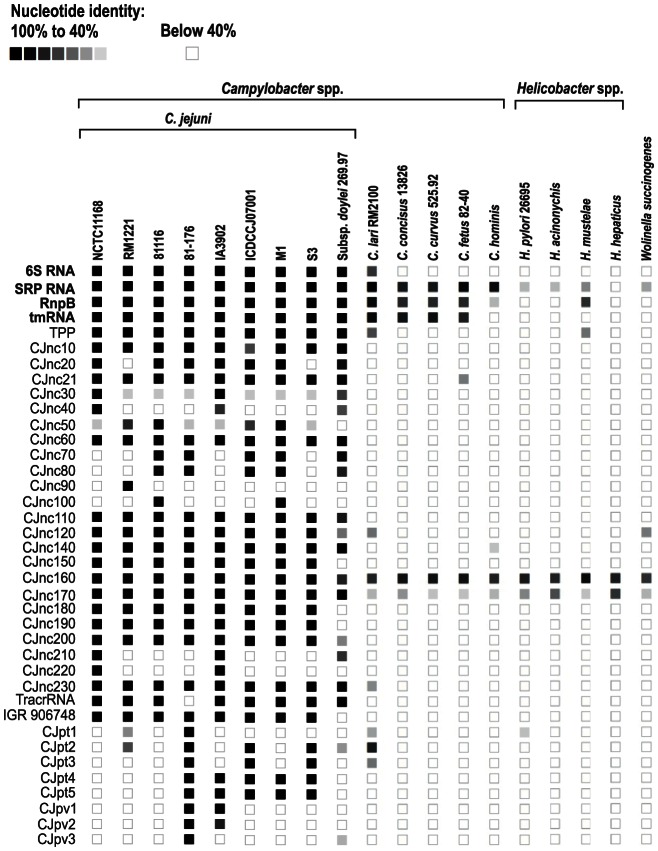
Conservation of *Campylobacter jejuni* sRNA candidates in Epsilonproteobacteria. Conservation of sRNA candidates in different *C. jejuni* strains and other representative Epsilonproteobacteria was analyzed by *BLAST* searches. The color intensity of the boxes represents the sequence identity to sRNAs of *C. jejuni* NCTC11168 or in case of the plasmid encoded sRNAs to strain 81–176. Identity values below 40% as well as the lack of an ortholog are symbolized by empty boxes. Housekeeping sRNAs are marked in bold.

Conservation analysis of our sRNA candidates showed that the majority of them are restricted to *Campylobacter jejuni* (Figure 5), indicating that they either have a specific regulatory function in this species or that their sequence conservation is not high enough to detect homologs by BLAST searches. Even the housekeeping RNAs (SRP RNA, tmRNA, RNase P RNA, and 6S RNA) are not conserved at the sequence level outside *Campylobacter* species. Several of the *C. jejuni* sRNA candidates, e.g., CJnc170 and CJnc190, are highly conserved in diverse strains, and could have a more general regulatory role within *C. jejuni*. In contrast, some sRNA candidates such as CJnc30 and CJnc80 or the plasmid-encoded sRNAs are found in only some of the strains, indicating strain-specific sRNA repertoires which might contribute to strain-specific regulation of gene expression.

### CRISPR loci in *Campylobacter*

**Figure 6:**
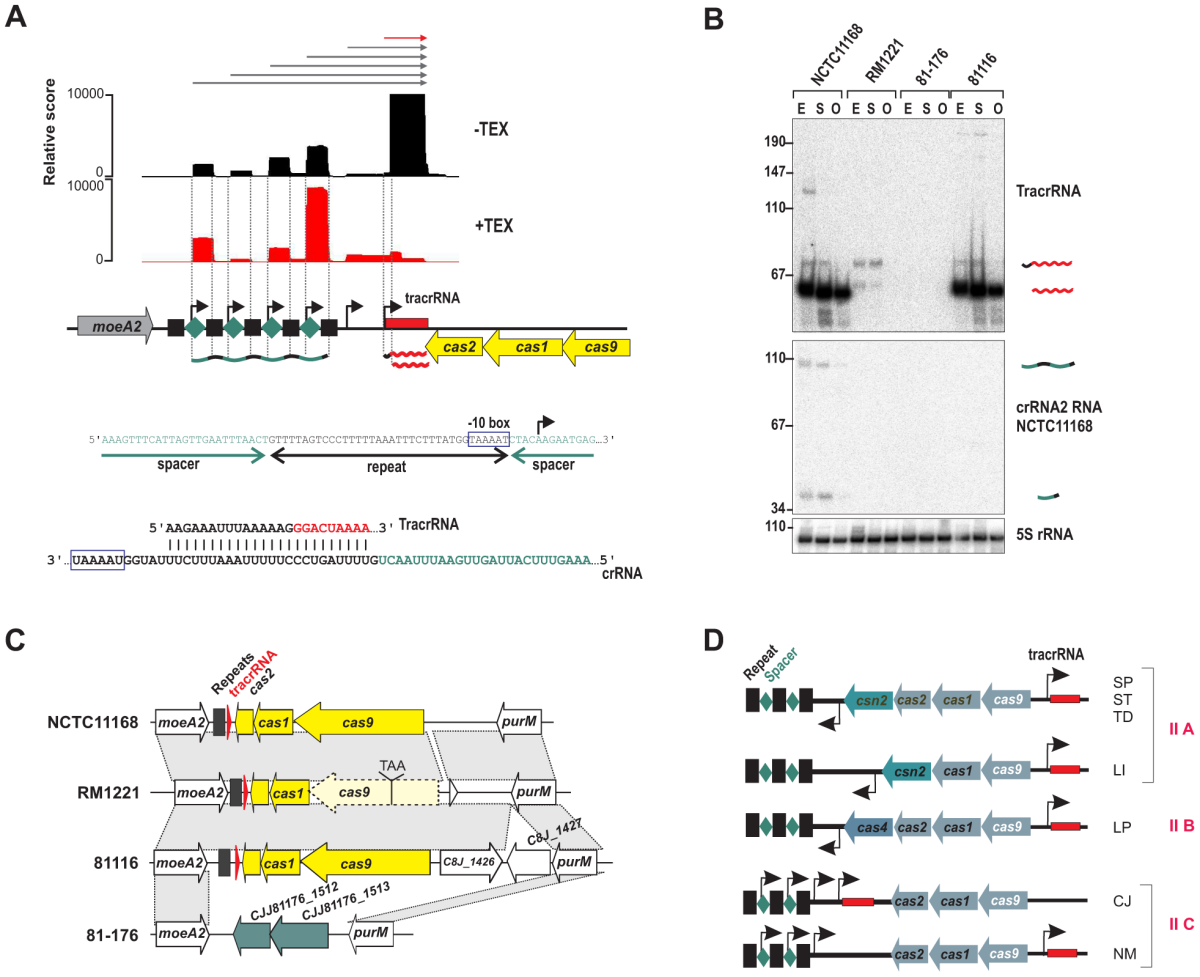
A minimal CRISPR in *Campylobacter jejuni*. (A) cDNAs reads of TEX−/+ libraries mapped to the CRISPR loci in *C. jejuni* NCTC11168. TSS are marked by black arrows. A −10 box for each crRNA is present in the 3′ part of the corresponding upstream located repeat (blue box). The lower panel shows the potential base-pairing of *C. jejuni* TracrRNA with the repeat sequence. (B) Northern blot analysis of TracrRNA and crRNAs. Total RNA (15 µg per lane) extracted from *C. jejuni* strains at mid-exponential (E), stationary (S), and overnight (O) growth phase was analyzed on a Northern blot using labeled DNA probes complementary to TracrRNA (two upper panels) and crRNA2 in NCTC11168 (see Table S14). Both the mature (∼62 nt) and processed (∼74 nt) TracrRNA forms as well as intermediate (∼103 nt) and mature (∼38 nt) crRNA forms for crRNA2 in NCTC11168 were detected. 5S rRNA served as loading control. (C) Conservation of CRISPR loci in different *Campylobacter jejuni* strains. In strain RM1221, *cas9* contains a premature-stop mutation. In strain 81–176 the whole CRISPR locus is replaced by two genes (green arrows) with low G/C-content. (D) Subclassification of type-II CRISPR-loci. *cas9*, *cas1*, and *cas2* are indicated with blue arrows and *tracrRNA* by a red box, respectively. TSS upstream of the repeat (black squares)-spacer (green diamonds) units in type-II A and B CRISPR loci [37], [87] or within each spacer in the case of the minimal type-II C CRISPR systems of *Campylobacter jejuni* (CJ) and *Neisseria meningitidis* (NM) are marked by black arrows. Representative species for each type-II class are listed (SP: *Streptococcus pyogenes*, ST: *S. thermophilus*, TD: *Treponema denticola*, LI: *Listeria innocua*, and LP: *Legionella pneumophila*).

One of the regions with highest numbers of cDNA reads in NCTC11168 and 811116 corresponded to the CRISPR (clustered regularly interspaced short palindromic repeats) locus. CRISPR loci are transcribed as precursors that are processed into mature crRNAs that together with Cas proteins silence invading foreign nucleic acids such as plasmids or phages [33], [34], [35]. Three of the four *C. jejuni* strains harbor a so-called type-II CRISPR/Cas system (Figure 6 and Figure S12 in Text S1), which requires a trans-encoded sRNA, TracrRNA, and the host factor RNase III for crRNA maturation [36], [37].

Our dRNA–seq analysis shows that the crRNAs and TracrRNA are actively transcribed and share a stretch of perfect complementarity in *C. jejuni* which allows for RNase III-dependent processing of the crRNAs. In line with this, we observe an accumulation of processed ∼38-nt spacer-repeat units and processed ∼62 nt TracrRNA (Figure 6B). Only weak CRISPR expression was detected in strain RM1221. Conservation analysis showed that the *cas9* gene, which is required for the stability of crRNAs and cleavage of target DNA [36], [38], carries a stop mutation in strain RM1221 leading to a truncated protein (Figure 6C). In strain 81–176 the CRISPR region is replaced by two genes with very low G/C content compared to the flanking genomic regions.

The RNase III/TracrRNA–dependent processing leads to a cleavage event within the repeat region of the crRNAs [36]. Surprisingly, the mature spacer-repeat units are enriched in our TEX-treated libraries, indicative of primary transcripts starting at position five within each spacer. Moreover, the 3′ end of each repeat ends with “GGTAAAAT” resembling an extended −10 box. Thus, *C. jejuni* apparently employs promoter sequences within each repeat to initiate transcription of the associated spacer unit, so that only one processing event mediated by RNase III and TracrRNA in the repeat sequence is required to generate the 3′ end of the mature crRNA. In line with this model, we observed accumulation of longer transcripts upon deletion of RNase III (Figure S13 in Text S1). Primer extension analysis confirmed that these longer transcripts start at the proposed TSS within each spacer and that most of the TracrRNA species are transcribed from these upstream promoters. Thus, in contrast to other type-II systems, where the crRNAs are transcribed together with a leader sequence from one upstream promoter, each repeat in the *C. jejuni* CRISPR carries its own promoter (Figure 6D).

## Discussion

Here we present the first comparative primary transcriptome analysis of four different *C. jejuni* strains including a novel automated TSS annotation method, which revealed strain-specific promoter usage and sRNA repertoires. The observed strain-specific transcriptional output reveals candidate genes which could contribute to phenotypic variation among strains or facilitate adaptation to different hosts or niches.

Previously, the generation of global TSS maps from RNA–seq data has mainly been performed on a manual or semi-automated basis [4], [39], [40], [41], [42], [43], [44], [45]. These strategies are typically very laborious and time-consuming with limited reproducibility and require even more effort for the comparison of transcriptomes from multiple conditions or strains. Therefore, we have developed a model-based TSS prediction method based on criteria used for manual TSS annotations. It provides detailed values (such as number of read starts, enrichment factor etc.) as well as classifications (pTSS, sTSS, asTSS, iTSS) for each TSS candidate which allows the user to review the underlying thresholds for TSS detection. Benchmarking of our TSS prediction method using the manually annotated TSS from *H. pylori* [4] showed that our approach achieved a sensitivity of 82% and a precision rate of 75%. Since our comparative approach allows for the integration of data sets from different strains or conditions, its performance can generally be increased by the inclusion and comparison of multiple data sets and replicates. Moreover, the TSS annotation will be further improved by adjusting the parameters to additional training sets based on experimental validation. Recently, Schmidtke *et al.* presented a fully automated approach for TSS annotation of a single genome based on read count data and a sophisticated statistical model, which calculates p-values for TSS candidates and thereby provides a confidence estimation for observing a TSS [46]. However, the specific properties of the TSS candidates, such as expression height and dRNA–seq enrichment factor, cannot be directly inferred from the resulting p-values. In future, a combination of both approaches would be promising especially for very weak TSS candidates. Furthermore, our automated TSS method also allows for a comparative TSS annotation among dRNA–seq libraries from multiple strains by the integration of the SuperGenome approach. Considering the rapidly increasing number of genome sequences as well as RNA–seq studies, our approach will facilitate a systematic TSS annotation among different strains or growth conditions and can also be adapted to the analyses of eukaryotic transcriptomes.

Comparative genomics of multiple *Campylobacter* strains or species provided the core genome of the genus and also revealed differences in genome content and structure which could support adaptation to different hosts [14], [15], [25], [47], [48]. Furthermore, comparison of genetically closely related *C. jejuni* strains isolated from different hosts indicated that *Campylobacter* uses high phenotypic flexibility and genetic microdiversity to reversibly adapt to changing environments [49]. In addition, variation in contingency loci contributes to rapid adaptation to novel hosts [50]. This genetic flexibility among strains might be supported by differences in transcription among strains and post-transcriptional regulation which we observed in our comparative dRNA–seq data. For this first comparative analysis we have selected widely used laboratory strains with available genome sequence. However, our comparative approach can be easily adapted to the analysis of multiple isolates from different hosts or isolation sources. For example, the use of our single-nucleotide resolution approach could help to understand the transcriptomic and phenotypic differences that were previously observed during the analysis of the genome-sequenced and original isolate of *C. jejuni* NCTC11168 [51].

Our comparative dRNA–seq approach allowed us to annotate between 1,905 and 2,167 TSS in the four *C. jejuni* strains. We observed that the majority of TSS are conserved among multiple strains, but we also found many examples where SNPs in promoter regions apparently resulted in strain-specific promoter usage. Thus, although some promoters are highly conserved and show almost perfect overlap to promoter consensus motifs - which would be indicative for active transcription - the respective genes are not necessarily expressed at the same level among strains. Therefore, comparative transcriptomics facilitates the identification of differences in the functional output from genomes which cannot be directly inferred from closely related DNA sequences.

SNPs can be adaptive and, thereby, lead to niche expansion [52]. In addition, point mutations in ORFs or rRNA genes can mediate antibiotics resistances in bacteria including *Campylobacter* [53]. In bacterial pathogens, a variety of pathoadaptive mutations have been described which can affect cell binding, host tissue tropism or virulence regulation [54], [55], [56], [57]. Furthermore, point mutations in ORFs could lead to truncated or non-functional proteins or changes in protein activity [58], [59], [60] and have also been shown to discriminate mRNA targets of bacterial sRNAs [61]. Even single regulatory genes or promoter inversions can be sufficient to alter bacterial host specificity [62], [63] and promoter SNPs have been associated with overproduction of virulence genes [64], [65]. Based on our global TSS maps, we identified several examples where SNPs disrupt conserved positions in promoter motifs recognized by the three sigma factors. Furthermore, we found several examples for disruptive SNPs in the A/T-rich upstream regions, indicating that this region is also required for transcription initiation in Epsilonproteobacteria. These promoters with SNPs might be good hints to genes which contribute to adaptation to different environments or hosts. For example, in strain 81116 we observed mutations in the promoter for monoheme cytochrome *c* and a molybdopterin oxidoreductase, which allow *C. jejuni* to use sulphite as a respiratory electron donor [32]. This indicates that strain 81116 has lost the ability to transcribe this operon and perhaps even lost the ability to respire sulphite (Figure 3). *C. jejuni* is unable to metabolize glucose since it lacks the enzyme phosphofructokinase. Instead, it has a complex branched respiratory chain and can utilize a variety of electron donors like formate, lactate, or sulphite. Sulphite respiration might also help *C. jejuni* to survive in sulphite-rich niches and foods, and might also be used for detoxification. Furthermore, a strain lacking the molybdopterin oxidoreductase has been shown to have a reduced ability to infect Caco-2 cells [66].

Since the SNPs in promoter regions could also interfere with binding of a transcriptional regulator and thereby affect transcription, such promoters with strain-specific expression patterns represent good candidates to fish for novel DNA-binding proteins. For example, a SNP in the Fur-binding site of the *sodB* promoter of certain *Helicobacter pylori* strains has been shown to affect direct binding of apo-Fur [67]. The global map of TSS and promoter SNPs may give hints as to how *C. jejuni* and other microbes with compact genomes and few transcription factors could adapt gene expression according to different environmental conditions.

Our comparative approach allowed us to annotate TSS by combining the dRNA–seq data from multiple strains. This approach improves the annotation accuracy of the individual strains by integrating the information from multiple transcriptomes and also reveals differences among strains. Previously, bacterial RNA–seq studies mainly focused on the analyses of single strains (reviewed in [2]) or were not strand-specific [68]. Two recent comparative transcriptome analyses using strand-specific cDNA sequencing mainly focused on the divergence of sRNA expression as well as long antisense RNAs [45], [69]. Apart from these studies, expression profiling of sRNAs in multiple strains has been reported only for a limited number of bacteria and was mainly based on Northern blot analysis [70], [71]. A global RNA–seq based comparison of regulatory elements including sRNAs was recently carried out between the two closely related species, *Escherichia coli* and *Klebsiella pneumoniae*, and revealed that the majority of orthologous operons were transcribed from different promoters [72].

Our global transcriptome maps revealed several candidates for conserved and strain-specific sRNAs. Small regulatory RNAs have been implicated as key regulators in metabolic pathways and during pathogenesis [73], [74]. The newly identified sRNAs could contribute to virulence gene regulation and host adaptation by modulating metabolic pathways which are important for host colonization in *C. jejuni* [19]. A study based on conventional, non strand-specific RNA–seq predicted five sRNAs in *C. jejuni* [24]. In our strand-specific dRNA–seq data, we observed expression for four of them (Figure S11 in Text S1). Moreover, a dRNA–seq study of *C. jejuni* NCTC11168 compared the transcriptome organization between *C. jejuni* and *H. pylori* [4] and identified around 20 trans-encoded sRNA candidates in *C. jejuni*, most of which are also detected in our study (I. Porcelli and A. van Vliet, personal communication). The majority of our sRNA candidates are expressed as independent transcripts. However, we also detected examples of processed sRNA species, which can be generated from the 3′ end of mRNAs. Recently, such 3′-end derived transcripts have been shown to stably associate with the RNA chaperone Hfq in *Salmonella* and to act as regulatory RNAs on *trans-*encoded mRNAs [75]. Since *Campylobacter* lacks Hfq, it will be interesting to see whether its sRNAs require a different RNA chaperone for their activity and stability or whether they act independently of an auxiliary protein. Moreover, future studies will be required to uncover the target genes and physiological roles of sRNAs in *Campylobacter*.

The most abundant sRNA in strain NCTC11168 corresponds to TracrRNA, which is required together with RNase III for maturation of CRISPR RNAs [36]. Due to the high variability of the spacer sequences, CRISPR loci have been used for strain genotyping including *Campylobacter* species [76]. Surprisingly, we found that the crRNAs in *C. jejuni* are transcribed from individual promoters within each repeat unit (Figure 6). In other prokaryotes, the crRNAs are transcribed from a leader sequence and several processing steps are required to generate the mature crRNAs. Therefore, the CRISPR locus of *Campylobacter* with its individual crRNA promoters and only three *cas* proteins represents a “minimal” system of the type-II subtype (Figure 6D) which requires only one processing event by RNase III within the repeats to generate the mature crRNAs. A similar CRISPR organization was also identified in *Neisseria menigitidis* (N. Heidrich and J. Vogel, personal communication). Furthermore, in strain RM1221, the crRNAs and TracrRNA were only weakly expressed probably due to a stop-mutation in *cas9*, while strain 81–176 completely lacks the CRISPR locus. Interestingly, these two strains carry prophages or plasmids, indicating that these horizontally acquired genetic elements could be mutually exclusive with an active CRISPR system. Conservation analysis in additional strains showed that strains with plasmids or integrated elements very often carry degenerated CRISPR loci (Figure S14 in Text S1). Moreover, it has recently been shown that ganglioside-like LOS structures of GBS-associated *C. jejuni* strains can confer efficient bacteriophage resistance and that the presence of sialyltransferases correlates significantly with an apparently non-functional CRISPR system [77]. Further studies of the influence of the CRISPR system on pathogenicity of *Campylobacter* will be required. Moreover, since components of type-II CRISPR systems have recently been adapted for genome editing in humans [78], [79], the minimal type-II systems might be useful for further improvements of such genome editing tools.

Overall our high-resolution transcriptome map revealed regulatory elements and their conservation in multiple *Campylobacter jejuni* strains on a genome-wide scale. The comparison of multiple strains improves annotation of transcriptome features such as TSS maps and reveals strain-specific TSS usage as well as sRNA repertoires. These strain-specific transcription patterns will provide new insights into genes which could promote phenotypic differences despite high conservation at the genome level. Our novel automated TSS annotation can easily be applied to a wider range of strains or conditions and may also be used for to the annotation of eukaryotic transcriptomes.

## Materials and Methods

### Bacterial strains and oligonucleotides

*Campylobacter jejuni* strains and DNA oligonucleotides used for cloning, as hybridization probes or for primer extension are listed in Tables S13 and S14, respectively.

### *Campylobacter jejuni* growth

Bacteria were grown on Müller-Hinton agar plates supplemented with 10 µg/ml vancomycin at 37°C under microaerobic conditions (10% CO_2_, 5% O_2_). For liquid cultures, a starter culture was inoculated with bacteria grown on plates to a final OD_600_ of 0.04 in 20 ml of Brucella broth (BB) medium including 10 µg/ml vancomycin and incubated overnight at 37°C under microaerobic atmosphere and an agitation of 140 rpm. The next day, 50 ml BB including 10 µg/ml vancomycin were inoculated to a final OD_600_ of 0.04 using the starter culture and incubated as described above. When the cultures reached mid-exponential (6.5 hrs; OD_600_ between ∼0.3–0.4), stationary (13 hrs; OD_600_ between ∼0.5–1) and overnight phase (29 hrs; OD_600_ between ∼0.5–1), culture volumes of cells corresponding to a total amount of 2, 4 and 8 OD_600_, respectively, were mixed with 0.2 volumes of stop-mix (95% EtOH and 5% phenol, V/V), frozen in liquid N_2_ and stored at −80°C until RNA extraction. The four strains varied in the final OD_600_ values that they reached at these growth phases, e.g., strain 81–176 showed highest OD_600_ values of up to ∼1.0 in stationary and overnight phase, whereas strain 81116 reached only OD_600_ values of ∼0.5. However, despite these differences in OD_600_ the harvested samples corresponded to similar growth phases along the growth curves of the four strains.

### RNA extraction and Northern blot

Frozen cell pellets were thawed on ice and resuspended in lysis solution containing 600 µl of 0.5 mg/ml lysozyme in TE buffer (pH 8.0) and 60 µl 10% SDS. Bacterial cells were lysed by incubating the samples for 1–2 minutes at 65°C. Afterwards, total RNA was extracted using the hot-phenol method described previously [80].

For Northern Blot analysis, 10 to 15 µg RNA was loaded per sample. After separation on 6% polyacrylamide (PAA) gels containing 7 M urea, RNA was transferred to Hybond-XL membranes, which were hybridized with γ32P-ATP end-labeled oligodeoxyribonucleotide probes indicated in Table S14. Candidate sRNAs with more than 50 reads (relative score) in untreated dRNA–seq libraries were probed on Northern blots.

### Construction of cDNA libraries for dRNA–seq and Illumina sequencing

For each of the four selected *C. jejuni* strains, NCTC1116, 81–176, 81116, and RM1221, dRNA–seq libraries were constructed from biological duplicates of RNA samples harvested at mid-log growth in BB.

Residual genomic DNA was removed from the total RNA isolated by DNase I treatment. For depletion of processed transcripts, equal amounts of *Campylobacter* RNA were incubated with Terminator™ 5′-phosphate-dependent exonuclease (TEX) (Epicentre #TER51020) as previously described [4].

Libraries for Solexa sequencing (HiSeq) of cDNA were constructed by *vertis* Biotechnology AG, Germany (http://www.vertis-biotech.com/), as described previously for eukaryotic microRNAs [81] but omitting the RNA size-fractionation step prior to cDNA synthesis. For details see Supplementary Methods in Text S1.

The resulting cDNA libraries were sequenced using a HiSeq 2000 machine (Illumina) in single-read mode. The raw, de-multiplexed reads as well as coverage files (see Supplementary Methods in Text S1) have been deposited in the National Center for Biotechnology Information's Gene Expression Omnibus [82] under the accession GSE38883.

For a detailed description of the read mapping, expression graph construction and normalization of expression graphs see Supplementary Methods in Text S1. In total, we sequenced between 2.3 to 5.5 Mio cDNA reads for each of the cDNA libraries which were subsequently mapped to the individual genome sequences (Table S1).

### Transcriptional start site (TSS) annotation and SuperGenome approach

A whole genome alignment of the four *C. jejuni* strains was computed with *Mauve* [83]. Based on this global alignment a common genomic coordinate system, the SuperGenome was defined into which all positional information can be projected that relates to the single genomes [29]. This resulted in a consensus sequence with the coordinates of the complete alignment and a mapping of each position of each single genome to a position in the alignment. Next, all genome-specific data (expression height graphs derived from mapped read data, genomic annotations, and sequences) were mapped to the common coordinate system.

Our automated TSS prediction approach, which uses this SuperGenome mapping for comparative analyses, consists of several steps: The initial detection of TSS in the single strains is based on the localization of positions, where a significant number of reads start. Thus, for each position i in the RNA–seq graph corresponding to the TEX+ library the algorithm calculates e(i)-e(i-1), where e(i) is the expression height at position i (Figure S15 in Text S1). In addition, the factor of height change is calculated, i.e. e(i)/e(i-1). To evaluate if the reads starting at this position are originating from primary transcripts, the enrichment factor is calculated as e_TEX+_(i)/e_TEX−_(i). For all positions where these values exceed the threshold (see Supplementary Material) a TSS candidate is annotated.

The TSS prediction procedure is applied to both replicates of each strain. TSS candidates, which are not detected in both replicates with a maximal positional difference of one nucleotide, are discarded. Afterwards, TSS candidates that are in close vicinity are grouped into a cluster and only the TSS candidate with the highest expression is kept. In the next step, the TSS candidates of each strain are mapped to the SuperGenome to assign each TSS to the corresponding TSS in the other strains. The final TSS annotations are then characterized on the SuperGenome level with respect to their occurrence in the different strains and in which strains they appear to be enriched. In the context of the individual strains the TSS are further classified according to their location relative to annotated genes. For this we used a similar classification scheme as previously described [4]. Thus, for each TSS it is decided if it is the *primary* or *secondary* TSS of a gene, if it is an *internal* TSS, an *antisense* TSS or if it cannot be assigned to one of these classes (*orphan*). A TSS is classified as *primary* or *secondary* if it is located ≤300 bp upstream of a gene. The TSS with the strongest expression considering all strains is classified as *primary*. All other TSS that are assigned to the same gene are classified as *secondary*. *Internal* TSS are located within an annotated gene on the sense strand and *antisense* TSS are located inside a gene or within ≤100 bp on the antisense strand. These assignments are indicated by a 1 in the respective column of Tables S4, S5, S6, S7, S8, S9. *Orphan* TSS, which are not in the vicinity of an annotated gene, are indicated by “0” in all four columns.

To validate our automated TSS detection we applied it to the previously generated dRNA–seq data of *Helicobacter pylori* grown under five different conditions [4]. In this study, we had manually annotated the TSS based on enrichment patterns in the TEX+ compared to TEX- libraries. We used these hand-curated TSS positions as benchmark and compared it to the results of the automated detection. We allowed a difference of up to one nucleotide when comparing an automatically detected TSS to a manually annotated TSS. With this threshold, the automated approach achieves a sensitivity of 82% and a precision rate of 75%. The parameters used for the TSS annotation in *C. jejuni* were selected according to this benchmarking with the manual TSS set of *H. pylori* (see also Supplementary Methods in Text S1).

### Promoter and RBS motifs detection and data visualization

To detect potential promoter motifs, sequence regions corresponding to 50 nt upstream of the TSS positions and the TSS position itself were scanned by *MEME* version 4.8.1 [84] with width parameters (fixed width of 45 nt as well as flexible widths). For the detection of ribosome binding site (RBS) motifs, the 5′UTR sequences of mRNAs were inspected by *MEME*. The 5′UTR length distributions were visualized using *R* and the *ggplot2* package.

### Cytochrome *c* oxidoreductase activity assay

The assay was adapted from Kappler *et al* [85]. The activity of the oxidoreductase enzyme was measured as the increase in cytochrome *c* absorbance at 550 nm when converted from the oxidized to the reduced form. Briefly, 2 µl cells (corresponding to 0.01 OD_600 nm_ in 10 mM Tris/HCl pH 8) were added to a freshly prepared mix containing the following: 45 µl 10 mM Tris/HCl pH 8 (with or without 2 mM sodium sulphite), 5 µl horse heart cytochrome *c* (10 mg/ml; #C2506, Sigma-Aldrich). Absorbance was measured after 15 minutes at 550 nm using an absorption coefficient of 20 mM^−1^cm^−1^ (spectrophotometer ND*-*1000*;* Peqlab). In the control sample, 2 µl of 10 mM Tris/HCl pH 8 was added instead of cells.

### Conservation analysis of sRNAs

To study the conservation of sRNAs in Epsilonproteobacteria, homologous sequences were searched with *blastn* (part of the BLAST+ package version 2.2.26 [86]; the word-size parameter was set to 10 nt). The number of identical nucleotides of the best hits of each sRNA candidate was divided by the total number of nucleotides of the query sRNA and multiplied by 100 to calculate the percentage value of conserved nucleotides. For visualization, conservation values ≥40% identity were translated into a gray scale and values below 40% were depicted as white boxes.

## Supporting Information

Table S1 Mapping statistics of *Campylobacter jejuni* dRNA–seq libraries. The table indicates the total number of sequenced cDNA reads considered in the analysis, the number of reads that were removed due to insufficient length (<12 nt) after poly(A)-tail clipping (before read mapping), the number of reads that were successfully mapped to the reference genomes or the pVir and pTet plasmids of strain 81–176 using *segemehl* (see Materials and Methods), the number of mappings (i.e. some reads map to different locations with the same score), and the number of uniquely mapped reads. For the number of mapped reads and number of uniquely mapped reads, the percentage values (relative to the total number of reads) are also listed.
(DOCX)

Table S2 Alignment statistics of the SuperGenome. The overall whole-genome alignment has 2,115,274 positions which are considered as the SuperGenome. 1,380,020 of these positions (∼65%) show 100% sequence identity in the four strains.
(DOCX)

Table S3 SuperGenome TSS table. The table contains information on positions and assigned classes of all annotated TSS. It lists all TSS that were detected in the SuperGenome (column “detected” = 1). In case a TSS was not detected in a certain strain the value of detected is “0”. If a TSS was mapped in more than one genome via the SuperGenome, there is one row for each genome the TSS was mapped to. Also, if the TSS is assigned to more than one class, there is one row for each class assignment and each associated gene. A detailed description of the individual columns of this table is listed as an extended legend in Text S1.
(XLSX)

Table S4 TSS detected in the individual *C. jejuni* strains. This table lists all TSS detected in the individual *C. jejuni* strains NCTC11168, RM1221, 81116, and 81–176 in separate Excel sheets. A detailed description of the individual columns of this table is listed as an extended legend in Text S1.
(XLSX)

Table S5 TSS detected on the pVir and pTet plasmids of *C. jejuni* 81–176. This table contains information on positions and class assignments of all TSS detected for the two plasmids of *C. jejuni* 81–176. We did not classify these TSS as being enriched or not. Instead the enrichment factor is provided in the table for each TSS, which allows for customized filtering. A detailed description of the individual columns of this table is listed as an extended legend in Text S1.
(XLS)

Table S6 TSS with previously described FliA and RpoN dependent promoters. This table lists genes with previously described FliA- [88] or RpoN-dependent promoters [89]. Conserved nucleotides, which are characteristic for each promoter type, are underlined.
(DOCX)

Table S7 TSS with a predicted FliA or RpoN motif. This table lists all TSS with a predicted FliA or RpoN motif as separate Excel sheets. The columns “MEME” and “Regular expression” indicate by which method the motif was detected.
(XLSX)

Table S8 Comparison of gene lengths for annotation correction. This table lists all ORFs of the four strains, their length, and whether a correction of the gene length is required. Orthologous genes are clustered. Only if orthologs in three or more strains were identified, which show a length difference, a correction attempt was performed.
(XLSX)

Table S9 Orthologs with corrected annotation. This table lists orthologs for the four strains for which the annotation was corrected based on comparison of ORF length among different strains.
(XLSX)

Table S10 5′UTR comparisons. For all possible combinations of the four strains, the CDS (coding sequence) length and 5′UTR length of orthologous genes were compared. Genes were considered as orthologous if they were best reciprocal hits in a BLAST search on DNA level (i.e. *blastn*) in two compared strains. The gene names, lengths of the CDS of the two genes, the type of the TSS (pTSS = both primary; sTSS = both secondary; mixed_pTSS_sTSS = one of two orthologs has a primary TSS, the other ortholog has a secondary TSS), and the lengths of the 5′UTR of each gene of a homolog pair as well as the comparison status of these (“equal” or “different”) are listed.
(XLS)

Table S11 sRNA candidates in the chromosomes of four *C. jejuni* strains. This table lists sRNA candidates in the chromosomes of *C. jejuni* strains NCTC11168, RM1221, 81116, and 81–176 in separate Excel sheets.
(XLSX)

Table S12 sRNA candidates on the pVir and pTet plasmids of *C. jejuni* 81–176. This table lists sRNA candidates in the pVir and pTet plasmids of strain 81–176.
(XLSX)

Table S13 Bacterial strains used in this study. Wild-type strains were kindly provided by the laboratories listed in this table.
(DOCX)

Table S14 DNA oligonucleotides used in this study. Sequences are given in 5′ → 3′ direction
(DOCX)

Text S1 Supplementary Material. This file contains Supplementary Methods, Figures S1-S15; extended legends for Tables S3, S4, and S5; and Supplementary References.
(PDF)
