# Supplementary material for: High-Resolution Transcriptome Maps Reveal Strain-Specific Regulatory Features of Multiple Campylobacter jejuni Isolates
Source: PLoS Genet. 2013 May 16;9(5):e1003495. doi: 10.1371/journal.pgen.1003495 (PMC3656092; doi:10.1371/journal.pgen.1003495)
Supplement: Table S1 — Mapping statistics of Campylobacter jejuni dRNA–seq libraries. The table indicates the total number of sequenced cDNA reads considered in the analysis, the number of reads that were removed due to insufficient length (<12 nt) after poly(A)-tail clipping (before read mapping), the number of reads that were successfully mapped to the reference genomes or the pVir and pTet plasmids of strain 81–176 using segemehl (see Materials and Methods), the number of mappings (i.e. some reads map to different locations with the same score), and the number of uniquely mapped reads. For the number of mapped reads and number of uniquely mapped reads, the percentage values (relative to the total number of reads) are also listed. (DOCX) [file pgen.1003495.s001.docx]

**Table S1. Mapping statistics of *Campylobacter jejuni* dRNA-seq libraries.** The table indicates the total number of sequenced cDNA reads considered in the analysis, the number of reads that were removed due to insufficient length (<12 nt) after poly(A)-tail clipping (before read mapping), the number of reads that were successfully mapped to the reference genomes or the pVir and pTet plasmids of strain 81-176 using *segemehl* (see Materials and Methods), the number of mappings (i.e. some reads map to different locations with the same score), and the number of uniquely mapped reads. For the number of mapped reads and number of uniquely mapped reads, the percentage values (relative to the total number of reads) are also listed.

|  | **NCTC11168 R1 -TEX** | **NCTC11168 R1 +TEX** | **NCTC11168 R2 -TEX** | **NCTC11168 R2 +TEX** | **RM1221**  **R1 -TEX** | **RM1221**  **R1 +TEX** | **RM1221**  **R2 -TEX** | **RM1221**  **R2 +TEX** |
| --- | --- | --- | --- | --- | --- | --- | --- | --- |
| **Total number of reads** | 2531653 | 3646875 | 2823393 | 4277090 | 5541367 | 3796307 | 4930476 | 3819843 |
| **Failed size filter after clipping** | 35426 | 108068 | 27541 | 167095 | 146201 | 38848 | 121493 | 83806 |
| **Total number of mapped reads** | 2463201 | 3373017 | 2749401 | 4014305 | 5344841 | 3696410 | 4742111 | 3679393 |
| **Total number of mappings** | 4778326 | 6051548 | 5642027 | 8734627 | 12716083 | 7651006 | 11133996 | 8154828 |
| **Uniquely mapped reads** | 1297582 | 2027267 | 1284067 | 1589563 | 1647729 | 1706330 | 1535896 | 1420358 |
| **% mappable reads** | 97.3 | 92.5 | 97.4 | 93.9 | 96.5 | 97.4 | 96.2 | 96.3 |
| **% of uniquely mapped reads** | 51.3 | 55.6 | 45.5 | 37.2 | 29.7 | 44.9 | 31.2 | 37.2 |
|  | | | | | | | | |
|  | **81116**  **R1 -TEX** | **81116**  **R1 +TEX** | **81116**  **R2 -TEX** | **81116**  **R2 +TEX** | **81-176**  **R1 -TEX** | **81-176**  **R1 +TEX** | **81-176**  **R2 -TEX** | **81-176**  **R2 +TEX** |
| **Total number of reads** | 4559972 | 3906174 | 3913148 | 4863622 | 4806372 | 2315766 | 3857841 | 3560672 |
| **Failed size filter after clipping** | 119288 | 25786 | 81165 | 61659 | 41887 | 27390 | 31904 | 41424 |
| **Total number of mapped reads** | 4393451 | 3775032 | 3797781 | 4698430 | 4709413 | 2242991 | 3785770 | 3441085 |
| **Total number of mappings** | 9702783 | 7072857 | 8724948 | 9624104 | 10844930 | 4066371 | 8640160 | 7547467 |
| **Uniquely mapped reads** | 1728726 | 2119356 | 1326766 | 2207903 | 1610393 | 1303871 | 1334936 | 1331049 |
| **% mappable reads** | 96.3 | 97 | 97 | 97 | 98.0 | 96.9 | 98.1 | 96.6 |
| **% of uniquely mapped reads** | 37.9 | 54 | 34 | 45 | 33.5 | 56.3 | 34.6 | 37.4 |
| **Mapped reads in plasmid pVir (NC_008770)** |  |  |  |  | 26271 | 30806 | 21861 | 45196 |
| **Mapped reads in chromosome (NC_008787)** |  |  |  |  | 4592849 | 2063904 | 3688606 | 3227514 |
| **Mapped reads in plasmid pTet (NC_008790)** |  |  |  |  | 90293 | 148281 | 75303 | 168375 |
| **Mappings in plasmid pVir (NC_008770)** |  |  |  |  | 27054 | 31433 | 22532 | 45902 |
| **Mappings in chromosome (NC_008787)** |  |  |  |  | 10727172 | 3886321 | 8542010 | 7332612 |
| **Mappings in plasmid pTet (NC_008790)** |  |  |  |  | 90704 | 148617 | 75618 | 168953 |
