# Supplementary material for: High-Resolution Transcriptome Maps Reveal Strain-Specific Regulatory Features of Multiple Campylobacter jejuni Isolates
Source: PLoS Genet. 2013 May 16;9(5):e1003495. doi: 10.1371/journal.pgen.1003495 (PMC3656092; doi:10.1371/journal.pgen.1003495)
Supplement: Table S2 — Alignment statistics of the SuperGenome. The overall whole-genome alignment has 2,115,274 positions which are considered as the SuperGenome. 1,380,020 of these positions (∼65%) show 100% sequence identity in the four strains. (DOCX) [file pgen.1003495.s002.docx]

**Table S2. Alignment statistics of the SuperGenome.** The overall whole-genome alignment has 2,115,274 positions which are considered as the SuperGenome. 1,380,020 of these positions (~65%) show 100% sequence identity in the four strains.

| **Strain** | **Accessionnr.** | **Genome length (nt)** | **Insertions (nt)** | **Deletions (nt)** | **Identical positions of genome size (%)** |
| --- | --- | --- | --- | --- | --- |
| **RM1221** | NC_003912 | 1777831 | 245093 | 93572 | 77.6 |
| **NCTC11168** | NC_002163 | 1641481 | 93305 | 78134 | 84.1 |
| **81-176** | NC_008787 | 1616554 | 54544 | 64300 | 85.4 |
| **81116** | NC_009839 | 1628115 | 96022 | 94217 | 85.8 |
| **SuperGenome** | -- | 2115274 | -- | -- | 65.2 |

The overall whole-genome alignment has 2,115,274 positions which are considered as the SuperGenome. 1,380,020 of these positions (~65%) show 100% sequence identity in the four strains.
