# Supplementary material for: High-Resolution Transcriptome Maps Reveal Strain-Specific Regulatory Features of Multiple Campylobacter jejuni Isolates
Source: PLoS Genet. 2013 May 16;9(5):e1003495. doi: 10.1371/journal.pgen.1003495 (PMC3656092; doi:10.1371/journal.pgen.1003495)
Supplement: Table S6 — TSS with previously described FliA and RpoN dependent promoters. This table lists genes with previously described FliA- [88] or RpoN-dependent promoters [89]. Conserved nucleotides, which are characteristic for each promoter type, are underlined. (DOCX) [file pgen.1003495.s006.docx]

**Table S6. TSS with previously described FliA and RpoN dependent promoters.** This table lists genes with previously described FliA- [88] or RpoN-dependent promoters [89]. Conserved nucleotides, which are characteristic for each promoter type, are underlined.

| **Gene** | **Name** | **-50 to +1 of TSS** | **TSS detected** |
| --- | --- | --- | --- |
| **FliA-dependent genes** TTT 10…12 CGAT [AT/TT/TA] | | | |
| Cj0547 | *flaG* | TTTTCTTTATAAGTGTTCATAAATTTATAAATTTGTCGATATAAGCTTTTA | Yes |
| Cj1339c | *flaA* | ATTTTATTGCTAAAGTATAAAATATTTTTTTGATTGCACGATATAGCATTT | Yes |
| Cj0045c | putative iron-binding protein | ATTCATTTGTCAAATTATTATTTTATTTTCAAAAAATACGATATAAAAATA | Yes |
| Cj0391c | hypothetical protein | GAATTTAATGTCCTTATAAATTTTTTAAGTCAAAAGTCGATATAAATTAAA | Yes |
| Cj0977 | hypothetical protein | TATACGCATTAAAATATAAATTCTTTTTATTTTTTGCCGATATTGAATTTT | Yes |
| Cj1464 | *flgM* | TTTAAAAGAAAGGGGTTAAGTTTTTTAAATTTAGGTCGATATGGTTTGTAG | No ^#^ |
| Cj1656c | hypothetical protein | AAGTTTAGTAAAAAATATTAAGTTTTAAAAATATCATCCGATTTAAATAAA | Yes |
| Cj0859c | hypothetical protein | AATTATTTTAAATTATAGTATATTTTTTTAAAATAATGTCGATTTAGTGTA | Yes |
| Cj1034c | adenylosuccinate lyase | TATTTTGTAAAAAAGTGTCAATCTTTATCTTATAAAGCCGATTATACACCT | No ^##^ |
| **RpoN-dependent genes** TGGNACANNNNNTGCTT | | |  |
| Cj1338c | *flaB* | AAATTTTTAATTAAACTAAAACTTGGAACACTTTTTGCTTTAATCTTTTCG | Yes |
| Cj1462 | *flgI* | TTTTTACTCTTCTTAACTCACTTGGAACACTTTTTGCTTGATCTATCATCA | Yes |
| Cj0528c | *flgB* | ATATTTTTATACAAAATAGTTAAATTGGAACAGTATTTGCTTGTTAATATT | Yes |
| Cj0887c | *flgL* | ATAAAATTATATAATTTAAATTTTTGGAACAGTTATTGCTTTTGTTTATTA | Yes |
| Cj0042 | *flgD* | TTGTTTTAGCGAAGTATTTTTAAATTGGAACACTTATTGCTTAAATAATAA | No ^#^ |
| Cj0697 | *flgG2* | TTATTTTTCTTTGTTTTTAAAAGTTGGAACACTCTTTGCTTTTATAGTTAT | No ^#^ |
| Cj0687c | *flgH* | TTTTCTCCTTGAATTTATAAATGTGGAACACTCTTTGCTTTTTCTAATTTT | No ^#^ |
| Cj1466 | *flgK* | GTAAATAAAATGTTTGATATCAATGATGGAACAAATAATGCTTATGGAGAT | No ^#^ |

^#^ Reads were detected in the 5’UTR region, but no enrichment was observed in the +TEX treated library.

^##^ Almost equal numbers of reads were observed in untreated and TEX-treated samples.
