# Supplementary material for: High-Resolution Transcriptome Maps Reveal Strain-Specific Regulatory Features of Multiple Campylobacter jejuni Isolates
Source: PLoS Genet. 2013 May 16;9(5):e1003495. doi: 10.1371/journal.pgen.1003495 (PMC3656092; doi:10.1371/journal.pgen.1003495)
Supplement: Table S13 — Bacterial strains used in this study. Wild-type strains were kindly provided by the laboratories listed in this table. (DOCX) [file pgen.1003495.s013.docx]

**Table S13. Bacterial strains used in this study.** Wild-type strains were kindly provided by the laboratories listed in this table.

| **Strain** | **Relevant markers/ genotype** | **Source** |
| --- | --- | --- |
| ***C. jejuni*** |  |  |
| **NCTC11168** |  | Arnoud van Vliet, Institute of Food Research, Norwich, UK |
| **81-176** |  | Patricia Guerry, Naval Medical Research Center, Silver Spring, MD, USA |
| **RM1221** |  | Dirk Hofreuter, Hannover Medical School, Hannover, Germany |
| **81116**  **NCTC11168** | *Δrnc::aphA3* | Steffen Backert, University College Dublin, Dublin, Ireland  This study (see Supplementary Methods in Text S1) |
