# Supplementary material for: High-Resolution Transcriptome Maps Reveal Strain-Specific Regulatory Features of Multiple Campylobacter jejuni Isolates
Source: PLoS Genet. 2013 May 16;9(5):e1003495. doi: 10.1371/journal.pgen.1003495 (PMC3656092; doi:10.1371/journal.pgen.1003495)
Supplement: Table S14 — DNA oligonucleotides used in this study. Sequences are given in 5′ → 3′ direction (DOCX) [file pgen.1003495.s014.docx]

**Table S14. DNA oligonucleotides used in this study.** Sequences are given in 5’ → 3’ direction.

| **Name** | **Sequence** | **Used for** |
| --- | --- | --- |
| CSO-0180 | CAAAAATGGGCAAATTGCAA | Northern blot probe CJnc10 |
| CSO-0181 | TTTTCTTATGAATGTTTTTCGGTTC | Northern blot probe CJnc140 |
| CSO-0182 | ATTTTCATGGGCTTGATTGCA | Northern blot probe CJnc170 |
| CSO-0183 | TCAGGATGAAATTTTTAACAAGTAGG | Northern blot probe CJnc30 |
| CSO-0184 | CGATGTGACATTTTATTCTAAAAACAC | Northern blot probe CJnc190 (full length) |
| CSO-0185 | GGAAATAGCCTAACCCAAACG | Northern blot probe CJnc190 (processed) |
| CSO-0187 | CATTTCGATCTCGTTGAGTTTTAAG | Northern blot probe CJnc20 |
| CSO-0188 | CCTTTTTATTCGTAGCCTAACTCTCT | Northern blot probe CJnc180 |
| CSO-0190 | GCTAGGTAATTTTTAAAGGCTGCA | Northern blot probe 6S RNA (CJnc130) |
| CSO-0191 | AAGTGCTCTGCTGTGTTCCCA | Northern blot probe SRP RNA |
| CSO-0192 | CTACTTTCCCCCTGCCAGTAA | Northern blot probe 5S rRNA |
| CSO-0193 | TTTTACTTTCATCATTTCATCCTTTG | Northern blot probe CJnc60 |
| CSO-0212 | CCTCGAATTTAAAAGGTGGG | Northern blot probe CJpt1 |
| CSO-0213 | TGGATATAATTTTGTGGGGTTAGC | Northern blot probe CJpt2 |
| CSO-0214 | TCAAATCACACTACAAGGAGGTG | Northern blot probe CJpt3 |
| CSO-0215 | AGCTTGACAAATAAAGGGTTAAGG | Northern blot probe CJpt4 |
| CSO-0216 | GTTTATTTAAGAATACCTTGCCGC | Northern blot probe CJpt5 |
| CSO-0217 | ACAAAGACAAAGGATTAAAGATGGA | Northern blot probe CJpv1 |
| CSO-0218 | CAAGTGTTTTATGGTTATGGGGT | Northern blot probe CJpv2 |
| CSO-0223 | AGCGGTTTTAGGGGATTGTAA | Northern blot probe Tracr RNA |
| CSO-0224 | AGGGACTAAAACAGTTAAATTCAACTAA | Northern blot probe crRNA2 (NCTC11168) |
| CSO-0240 | TATCACTGTCCTGTTTGTTATGAG | Sense aligo binds to upstream of upstream region of *rnc* |
| CSO-0241 | TCCTAGTTAGTCACCCGGGTACAACTTTTATGGGTTAAAGCATGT | Antisense oligo for amplification of upstream region of *rnc*; incl. complementary region to HPK1 |
| CSO-0242 | GTGAAAAACAATGAAACACATTG | Sense oligo for amplification of upstream region of *rnc* |
| CSO-0243 | TCTTCTTCTTTCAAATTTGATACAA | Antisense oligo for amplification of downstream region of *rnc* |
| CSO-0244 | AATTGTTTTAGTACCTGGAGGGAATACAAAAAAGAAGCACAGCAAAT | Sense oligo for amplification of downstream region of *rnc* incl. complementary region to entire HPK2 |
| CSO-0270 | AGGGACTAAAACATTTAAGTCCATTT | Northern blot probe crRNA4 (NCTC11168) |
| CSO-0296 | CTAAAAGGGACGAACAACCATC | Northern blot probe CJas_Cj0363c |
| CSO-0297 | GAAAGTATGTTTCCACAAGCCTTA | Northern blot probe CJas_Cj0566 |
| CSO-0298 | CGCTTAGGTGCTTATTATCAATTTC | Northern blot probe CJas_Cj0704 |
| CSO-0299 | ACTCTTGGCGGAAATCCAC | Northern blot probe CJas_Cj1667c |
| CSO-0300 | ATGCCCAGCCTTGATATAAGTC | Northern blot probe CJas_Cj0168c |
| CSO-0302 | CCATCTCTTTCTACCACCGG | Northern blot probe CJnc100 |
| CSO-0303 | AGATCATTGGATTTTTAGTTCTTGTC | Northern blot probe CJnc80 |
| CSO-0397 | CCTACGGCTAAATTGTGAAACC | Northern blot probe CJnc90 |
| CSO-0398 | CTTGCAACCGAAAGAGTAGCC | Northern blot probe CJnc70 |
| CSO-0399 | GCCAATTCTTCGCTAAACTTTCA | Northern blot probe CJpv3 |
| CSO-0496 | GCACATCAGTTTCATTTTTTCTCC | Northern blot probe CJnc110 |
| CSO-0497 | GTTAGATGGAATGTTTGTCTTTAGCAG | Northern blot probe RnpB |
| CSO-0498 | TTGGGCTTTTTACGCTTTGC | Northern blot probe tmRNA |
| CSO-0514 | CGCTAAAACAGCGAAAAAAATAGC | Northern blot probe CJas_CJJ81176_1020 |
| CSO-0525 | AAGCCCTACACGACCTACACG | Northern blot probe CJnc22 |
| CSO-0526 | CCCATTCTAAACGACGCGAC | Northern blot probe CJnc21 |
| CSO-0527 | TACATCATTCCGCCCATGC | Northern blot probe CJnc120 |
| CSO-0536 | AGCTTTATTTACAAGAGCATCTCTGAT | Northern blot probe CJnc11 |
| CSO-0537 | AGGCAGTTCAAGCCTAAGCC | Northern blot probe CJnc230 |
| HPK1 | GTACCCGGGTGACTAACTAGG | Sense oligo for amplification of *aphA3* cassette |
| HPK2 | TATTCCCTCCAGGTACTAAAACA | Antisense oligo for amplification of *aphA3* cassette |
