# Supplementary material for: High-Resolution Transcriptome Maps Reveal Strain-Specific Regulatory Features of Multiple Campylobacter jejuni Isolates
Source: PLoS Genet. 2013 May 16;9(5):e1003495. doi: 10.1371/journal.pgen.1003495 (PMC3656092; doi:10.1371/journal.pgen.1003495)
Supplement: Text S1 — Supplementary Material. This file contains Supplementary Methods, Figures S1-S15; extended legends for Tables S3, S4, and S5; and Supplementary References. (PDF) [file pgen.1003495.s015.pdf]

# Text S1

# Supplementary Material

## High-resolution transcriptome maps reveal strain-specific regulatory features of multiple *Campylobacter jejuni* isolates

**This supplement contains:**


| Supplementary | Page |
|---|---|
| Methods | 2-6 |
| Figures S1-S15 | 7-26 |
| Legends Tables S3-S5 | 28-30 |
| References | 31 |

## Supplementary Methods

### Phylogenetic tree of Epsilonproteobacteria

To determine the phylogenetic relationship between the analyzed *Campylobacter jejuni* strains and close relatives, the sequences of twelve conserved proteins (namely Ffh, FusA, GyrB, InfB, LepA, PyrG, RplB, RpsE, RpsH, RpsK, TopA, and Tuf), which were previously proposed for phylogenetic analyses [1], of *Campylobacter jejuni* NCTC11168 were retrieved. For each protein, the ortholog in 21 selected species (*Helicobacter acinonychis* str. Sheeba, *Helicobacter felis* ATCC 49179, *Helicobacter hepaticus* ATCC 51449, *Helicobacter mustelae* 12198, *Helicobacter pylori* 26695, *Wolinella succinogenes, Arcobacter butzleri* RM4018, *Campylobacter concisus* 13826, *Campylobacter curvus* 525.92, *Campylobacter fetus* subsp. fetus 82-40, *Campylobacter hominis* ATCC BAA-381, *Campylobacter jejuni* subsp. *doylei* 269.97, *Campylobacter lari* RM2100, and *Campylobacter jejuni* strains CG8421, RM1221, 81116, 81-176, IA3902, ICDCCJ07001, M1, and S3) was identified by *BLAST* searches [2] of the protein sequence against all protein sequences of the considered organism. The best hit of the *BLAST* search was considered as the ortholog. This search led to twelve protein collections with 22 members each. For each group of orthologs, a multiple sequence alignment was generated using the program *muscle* [3]. These alignments were concatenated to one single alignment with a total length of 6,376 amino acid columns. This alignment was filtered by *gblocks* [4] to remove poorly aligned positions and variant regions resulting in an alignment of 4442 positions. Based on this hybrid alignment, 4,500 bootstrapped trees were generated by PhyML 3.0 [5] applying the LG matrix [6]. A consensus tree was generated using program *consense* from the Phylip package (Felsenstein, J. 1993. PHYLIP (Phylogeny Inference Package) version 3.5c; distributed by the author (Department of Genetics, University of Washington, Seattle). In the next step *PhyML* estimated the branch lengths based on the topology of consensus tree and the previously described sequence alignment. The bootstrap values and branch lengths were merged into one file in the Newick format using *clann* [7]. For an easier comparison, the branch lengths were calculated as $\log_e$ values and normalized. Visualization and manual re-rooting was done in the webtool *iTOL* [8].

### Construction of dRNA-seq libraries

cDNA libraries for Solexa sequencing (HiSeq) were constructed by *vertis* Biotechnologie AG, Germany (http://www.vertis-biotech.com/) as described previously for eukaryotic microRNA [9] but omitting the RNA size-fractionation step prior to cDNA synthesis. In brief, equal amounts of RNA samples were poly(A)-tailed using poly(A) polymerase. Then, the 5'PPP structures were removed using tobacco acid pyrophosphatase (TAP). Afterwards, an RNA adapter was ligated to the 5´-phosphate of the RNA. First-strand cDNA was synthesis by an oligo(dT)-adapter primer and the M-MLV reverse transcriptase. In a PCR-based amplification step using a high fidelity

DNA polymerase the cDNA concentration was increased to 20-30 ng/µl. A library-specific barcode for multiplex sequencing was part of a 3'-sequencing adapter. The following adapter sequences flank the cDNA inserts:

TrueSeq_Sense_primer

5′AATGATACGGCGACCACCGAGATCTACACTCTTTCCCTACACGACGCTCTTCCGATCT-3'

TrueSeq_Antisense_ NNNNNN_primer (NNNNNN = 6n barcode for multiplexing)

5'-CAAGCAGAAGACGGCATACGAGAT-NNNNNN-GTGACTGGAGTTCAGACGTGTGCTCTTCCGATC(dT25)-3'

The Agencourt AMPure XP kit (Beckman Coulter Genomics) was used to purify the DNA which was analyzed by capillary electrophoresis afterwards.

**Read mapping and coverage plot construction**

To assure a high sequence quality, the Illumina reads in FASTQ format were trimmed with a cut-off phred score of 20 by the program *fastq_quality_trimmer* from FASTX toolkit version 0.0.13. After trimming, poly(A)-tail sequences were removed and a size filtering step was applied in which sequences shorter than 12 nt were eliminated. The collections of remaining reads were mapped to the respective reference genomes using *segemehl* [10]. Coverage plots representing the number of mapped reads per nucleotide were generated based on the mapped reads and visualized in the *Integrated Genome Browser* [11]. Each graph was normalized to the number of reads that could be mapped from the respective library. To restore the original data range, each graph was then multiplied by the minimum number of mapped reads calculated over all libraries.

**Normalization of expression graphs**

Prior to the comparative analysis, the expression graph data that resulted from the read mapping were further normalized. A percentile normalization step was applied to normalize the TEX+ graphs. To this end, the $90^{th}$ percentile of all data values was calculated for each TEX+ graph. This value was then used to normalize the TEX+ graph as well as the respective TEX- graph. Thus, the relative differences between each TEX+ and TEX- graph were not changed in this normalization step. Again, all graphs were multiplied with the overall lowest value to restore the original data range. To account for different enrichment rates, a third normalization step was applied. During this step, prediction of TSS candidates was performed for each replicate of each strain. These candidates were then used to determine the median enrichment factor for each TEX+/- library pair. Using these medians all TEX- libraries were then normalized against the library with the strongest enrichment.

**Transcriptional start site (TSS) annotation**

Based on the normalized expression graphs, our TSS annotation approach was first applied to each strain individually. The parameters used for the TSS annotation were adjusted based on our *H. pylori* dRNA-seq training data set [12] for which TSS have been annotated manually and, thus, could be used for comparison. Based on this criterion, we set the threshold for the minimum flank height to 3.45 (see Figure S15). If the TSS candidate reaches this threshold in at least one strain, the threshold is decreased for the other strains to 1.15. The threshold for the minimum factor of height change is set to 2.0, which, if reached in at least one strain, drops to 1.5 for the others. We consider a TSS candidate to be enriched in a strain if the respective enrichment factor is at least 2.0. A TSS candidate has to be enriched in at least one strain and is discarded otherwise. If a TSS candidate is not enriched in a strain but still reaches the other thresholds it is only indicated as "detected". However, a TSS candidate can only be labeled as detected in a strain if its enrichment factor is above 0.66. Otherwise we consider it to be a processing site.

**Identification/determination of orthologs**

Clusters of orthologous genes among the four *Campylobacter jejuni* strains were generated as follows: 1) Each gene was *blasted* [2] against all genes of the other three strains with default parameters, except for using a word length of 10. 2) Orthologous gene pairs for each pair of strains were created by searching for best reciprocal hits of genes. Only *BLAST* matches with an e-value of 0.01 or below and an alignment length of at least 60% of the query length were taken into account. 3) These pairs of best reciprocal hits were merged into clusters of orthologs by grouping pairs that share one gene. In total there were 1,454 clusters with 4 orthologs, 78 clusters with 3 orthologs, 132 clusters with 2 orthologs and 813 genes without any ortholog (Table S8).

**Consensus based correction of gene annotations**

The length comparison of orthologous ORFs in the four strains revealed inconsistencies in the annotations for several genes. Some genes were longer or shorter than their orthologs, i.e. differed at their 5' end, although the surrounding genomic region was conserved or at least very similar. Due to these annotation differences, a consensus based correction of such annotations was performed in which only aberrations of the 5' end were tried to be corrected. To determine if the 5' end of a gene with aberrant length was similar to the sequence of its orthologs, the first 10 nucleotides of each ortholog-group member were extracted and the Hamming distances of the start sequence of the aberrant gene and the start sequence of each of its orthologs was calculated. If the Hamming distance exceeded the value 1, the 5' end was assumed to be

different. In the case that there were three or two orthologs with the same and one with an aberrant sequence length or in the case of two with the same and two with aberrant lengths, the length of the majority was taken as target value for the aberrant ones. Based on the genomic sequences the annotation of the aberrant gene was extended or shortened to the target length. If the sequence of the modified annotation started with a potential start codon (ATG, TTG or GTG) the correction was accepted. In the case that there were two pairs of genes with the same sequence length, we tried to correct the length of both members of each pair to the length of the other pair as done above. If both pairs could be shortened or extended to the length of the other and fulfilled the requirement of having a valid start codon, manual inspection of the ortholog group was used to decide how to perform the correction. If all 3 or 4 members of a group of orthologs differed in length, or if there were only two orthologs in a group, no correction attempt was performed. The corrected annotations are listed in Table S9.

**Comparison of 5'UTR lengths of orthologs**

The 5' UTR lengths of orthologous genes were compared for pairs of strains (Table S10) and visualized as scatter plots (Figure S5B). For this purpose, the types of the originating TSS (primary or secondary) of the 5'UTRs were discriminated. If one or both genes of an ortholog pair had more than one TSS of the same type, 5'UTR pairs with the minimal distances were selected from all possible constellations. In case a 5' UTR had no partner of the same length and same type but a partner of same length and different type, the mixed type comparison was considered for the plotting.

**Searches of alternative sigma factor promoter sequences using regular expressions**

To find genes that contain promoter sequences of the alternative sigma factors, $\sigma^{28}$ and $\sigma^{54}$, that were not detected during the MEME motif generation, the -50 to +1 sequences for each TSS were scanned with regular expressions: "TGG.ACA.[5]TGCTT" for $\sigma^{54}$ and "TTT.[10,12]CGAT(AT|TT|TA)" for $\sigma^{28}$ using the UNIX tool *grep* with the parameter "-E". In the SuperGenome, 172 sequences had hits for the $\sigma^{28}$ pattern and 36 hits for the $\sigma^{54}$ pattern (see Table S7).

**Construction of a *rnc* deletion mutant**

A *C. jejuni* NCTC11168 Δ*rnc* (Cj1635c) deletion mutant (Δ*rnc::aphA-3*) was generated by homologous recombination using a PCR-amplified non-polar cassette carrying the *aphA*-3 kanamycin resistance gene [13] flanked by ~500 bp homology regions up- and downstream of the *rnc* gene. The upstream and downstream flanking regions of the *rnc* gene were amplified from genomic DNA of *C. jejuni* NCTC11168 using Phusion Polymerase (Finnzymes) and the primer sets CSO-0241/CSO-0242 and CSO-0243/CSO-0244, respectively (Table S22). CSO-0241

and CSO-2044 contain homologous sequences to the 5' or 3' end of a non-polar *aphA-3*, which was amplified from genomic DNA of *H. pylori* carrying a deletion mutant of the sRNA HPnc5490 (*ΔHPnc5490::aphA-3)* using the primer set HPK1/HPK2 [12]. An overlap PCR strategy was used to create a single fragment by joining the above three fragments with *aphA-3* in between using the primer set CSO-0242/CSO-0243 as previously described but using Phusion Polymerase (Finnzymes) [14]. The overlap PCR was run on a 1% agarose gel and the correct fragment was excised and purified from the gel.

1 µg of the final construct was then electroporated into freshly prepared electrocompetent *C. jejuni* NCTC11168 as previously described [15]. The cells were left overnight to recover on non-selective Müller-Hinton (MH) plates containing 10 µg/ml vancomycin. The next day, cells were restreaked on selective MH plates containing 10 µg/ml vancomycin and 50 µg/ml kanamycin. Colonies observed on the selective plate after 4 days were restreaked for genomic DNA isolation using the NucleoSpin Plasmid kit (Macherey-Nagel), which allowed us to isolate genomic DNA of *Campylobacter.* PCR amplification using the isolated genomic DNA and primer set CSO-0240 (binds upstream of CSO-0242) and CSO-0023 (antisense to *aphA-3*) were used to confirm correct integration of the cassette and deletion of the *rnc* gene.

Moreover, deletion of the *rnc* gene was confirmed by analysis of total RNA of samples harvested at log phase from *Campylobacter jejuni* wild-type and Δ*rnc* strains on a 1% agarose/TBE gel. As expected, deletion of RNase III leads to loss of processing of the 23S rRNA precursor transcript (data not shown).

**Primer extension**

5 µg of DNase I digested RNA extracted from the exponential phase of *C. jejuni* NCTC11168 wild-type and Δ*rnc* strains was concentrated in 5.5 µl water. 1µl of $\gamma^{32}$P-ATP end-labeled oligodeoxyribonucleotide probe CSO-0223 (hybridizes to the 3' end of TracrRNA) or CSO-0270 (hybridizes to crRNA4 in NCTC11168) were mixed with the two RNA samples and incubated at 80°C. The temperature was gradually shifted to 42°C over a period of 1 hr. Each of the samples was then mixed with 1 µl 10 mM dNTPs, 2 µl 5xAMV RT buffer and 0.5 µl AMV RT 20 u/µl (Fermentas, #EP0641) and incubated further at 42°C for 1 hr. Samples were denatured for 3 min at 95 °C and half of the reaction was separated on a 6% polyacrylamide/8.3 M urea sequencing gel in 1X TBE. Sequencing ladders were generated according to the manufacturer's instructions by SequiTherm EXCEL™ II DNA Sequencing Kit (Epicentre Biotechnologies) using the same primers and PCR-amplified CRISPR locus from NCTC11168 (Oligos CSO-0253 and CSO-0258) as template. 2 µl of the sequencing reactions were loaded on the same gel as reference.

## Supplementary Figures

### Figure S1

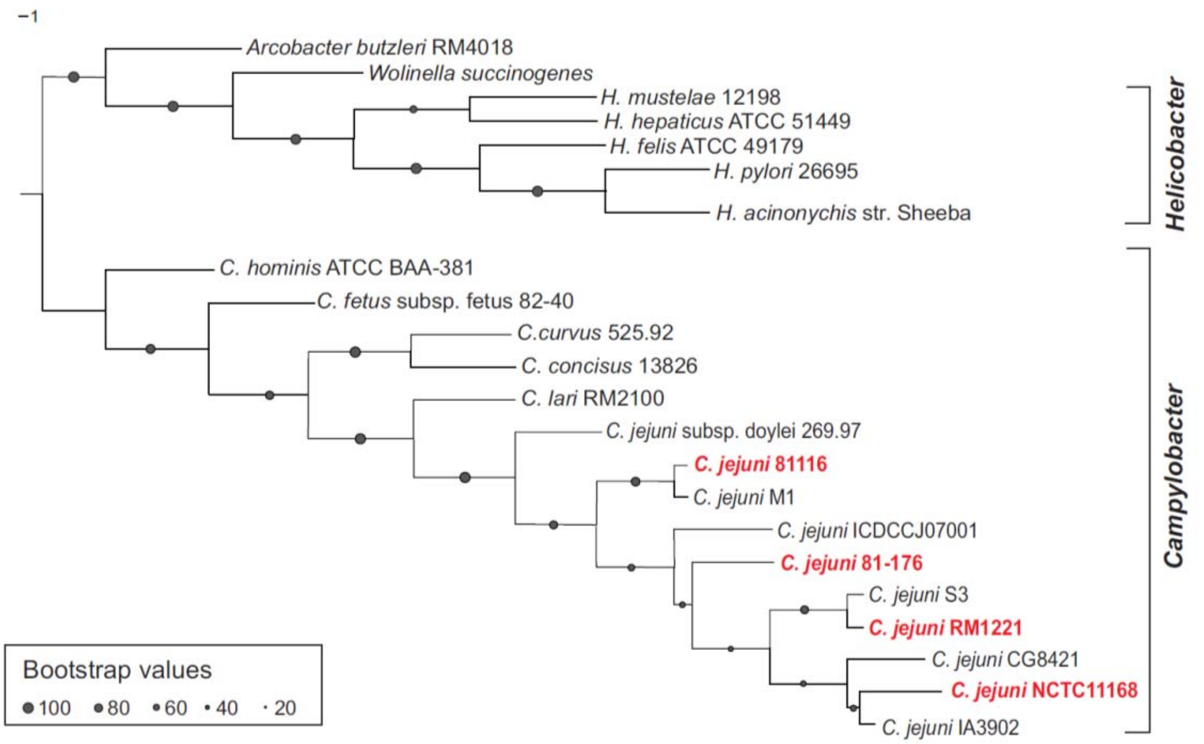


**Phylogenetic tree of representative members of the Epsilonproteobacterial lineage.** The phylogenetic tree was calculated based on concatenated sequence alignments of 12 highly conserved proteins (Ffh, FusA, GyrB, InfB, LepA, PyrG, RplB, RpsE, RpsH, RpsK, TopA, and Tuf). Bootstrap values are based on 4,500 trees that were combined to the presented consensus tree. The four strains analyzed in this study are highlighted in red.

**Figure S2**

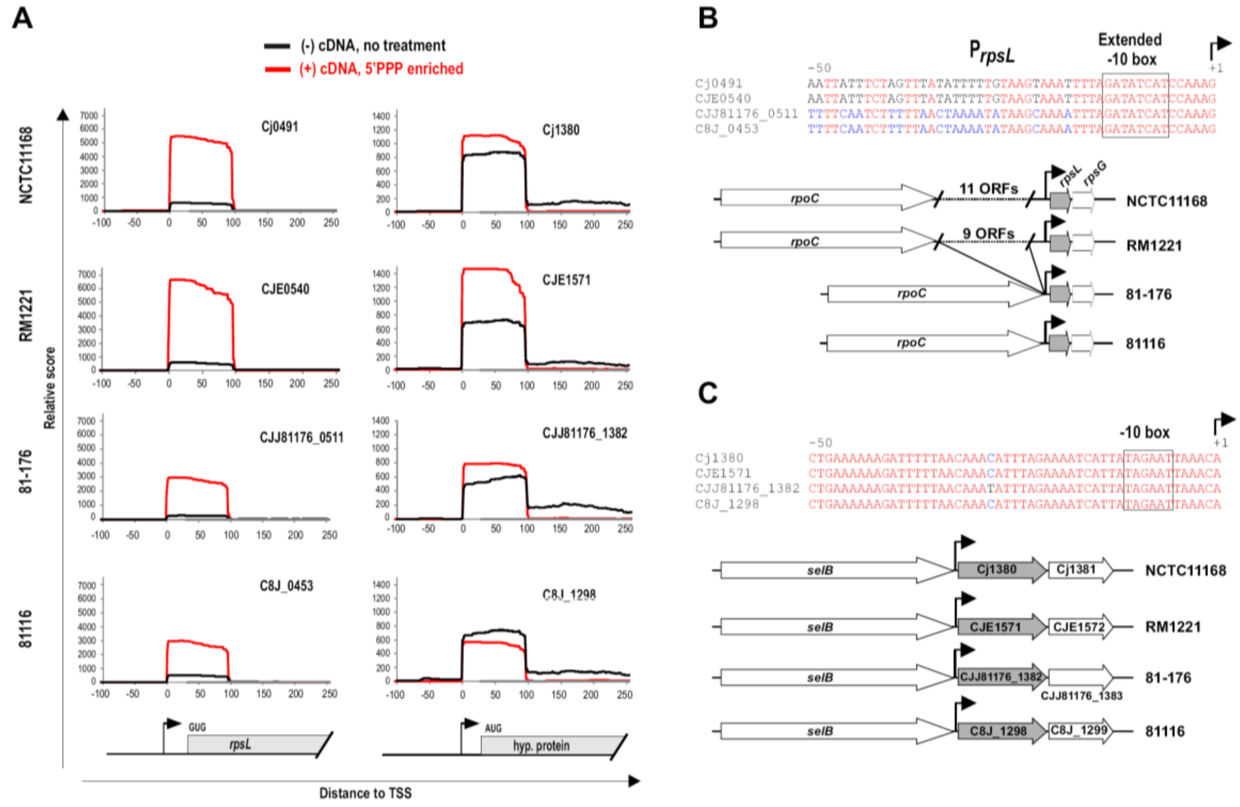


**Comparative annotation of transcriptional start sites (TSS) based on differential RNA-seq. (A)** Schematic drawing of cDNA enrichment patterns at primary 5' ends of *rpsL* mRNAs, encoding ribosomal protein S12 (left), and mRNAs of Cj1380 homologs, encoding a gene of unknown function (right). Exonuclease treatment (TEX+, red curve) shifts the cDNAs towards the nuclease-protected 5'-end, yielding a sawtooth-like profile with an elevated sharp 5' flank that corresponds to the TSS. A clear enrichment in the TEX+ libraries was observed at the TSS of *rpsL* in all four strains. In contrast, a clear enrichment at the TSS of homologs of Cj1380 was only observed for CJE1571 and a weak enrichment for Cj1380 and CJJ81176_1382. The cDNA coverage at the TSS of C8J_1298 is even higher in the TEX- library compared to the TEX+ library, which is rather indicative for a processing site. **(B)** Nucleotide alignment of the *rpsL* promoter (-50 to +1 according to the TSS) and genomic location of *rpsL* in the four analyzed *C. jejuni* strains. Based on sequence conservation solely, it would be unclear whether the *rpsL* promoter is functional in strains NCTC11168 and RM1221, since 11 and 9 ORFs, respectively, are inserted directly upstream of its -10 box and might disrupt the promoter. However, the enrichment patterns in the dRNA-seq data on the left indicate active promoters in all four strains. **(C)** Nucleotide alignment of the promoter regions and genomic location of Cj1380 homologs in the four analyzed *C. jejuni* strains.

**Figure S3**

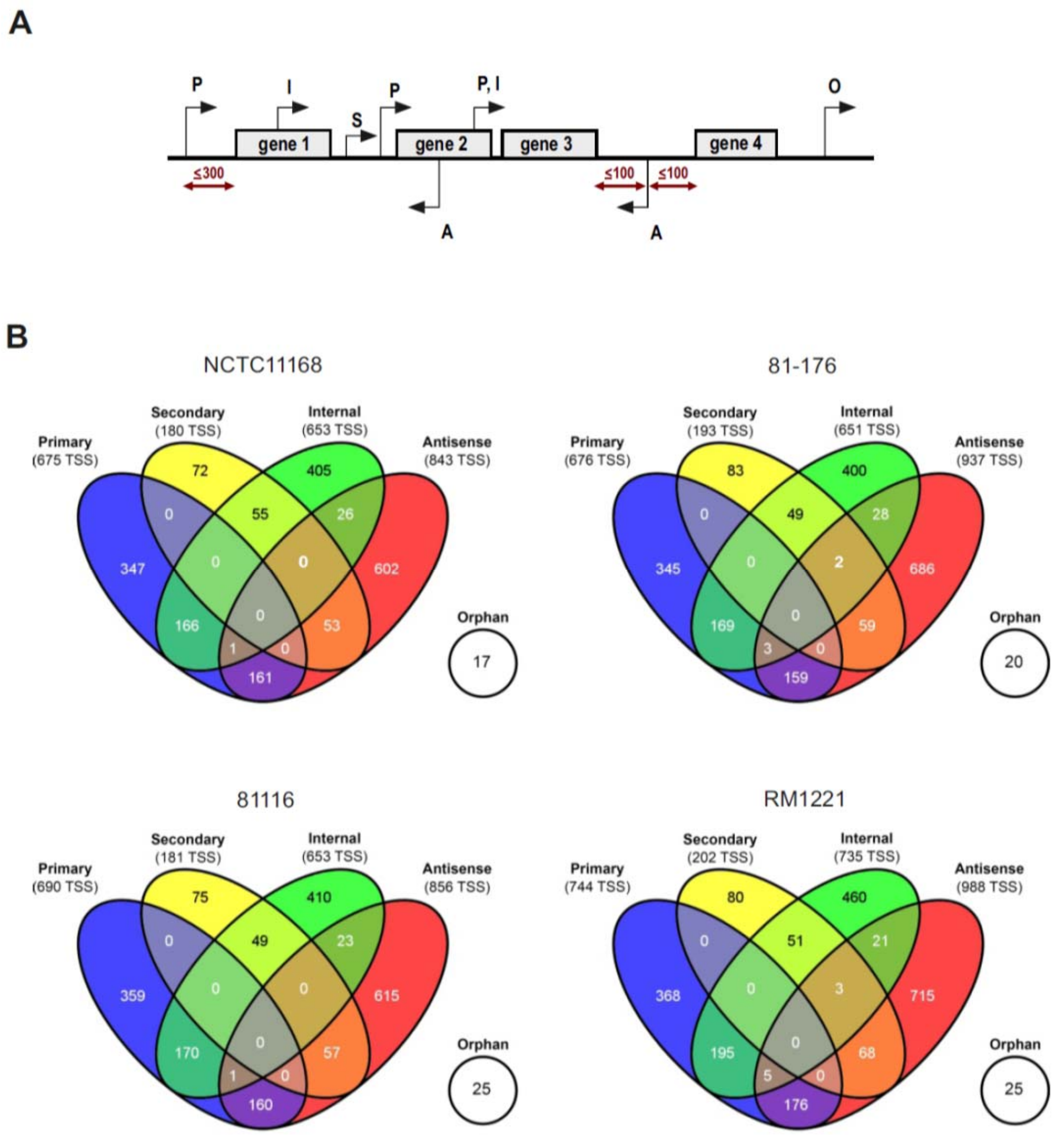


**TSS classifications for the four *C. jejuni* strains. (A)** Representation of TSS classifications based on expression strength and genomic context: primary (P), secondary (S), internal (I), antisense (A), or orphan (O). Cut-offs for the distances to flanking genes are indicated above the red arrows. **(B)** The Venn diagrams indicate the overlap between TSS classes for the four individual strains. Many TSS are assigned to more than one class. For example in NCTC11168, 167 of the 675 *primary* TSS (~25%) are also classified as *internal* and 162 (~24%) are also classified as *antisense*. In 81-176, 172 of the 676 *primary* TSS (~25%) are also classified as *internal* and 162 (~24%) are also classified as *antisense*. In 81116, 171 of the 690 *primary* TSS (~25%) are also classified as *internal* and 161 (~23%) are also classified as *antisense*. In RM1221, 200 of the 744 *primary* TSS (~27%) are also classified as *internal* and 181 (~24%) are also classified as *antisense*. Venn diagrams were generated by VENNY (http://bioinfogp.cnb.csic.es/tools/venny/index.html).

**Figure S4**

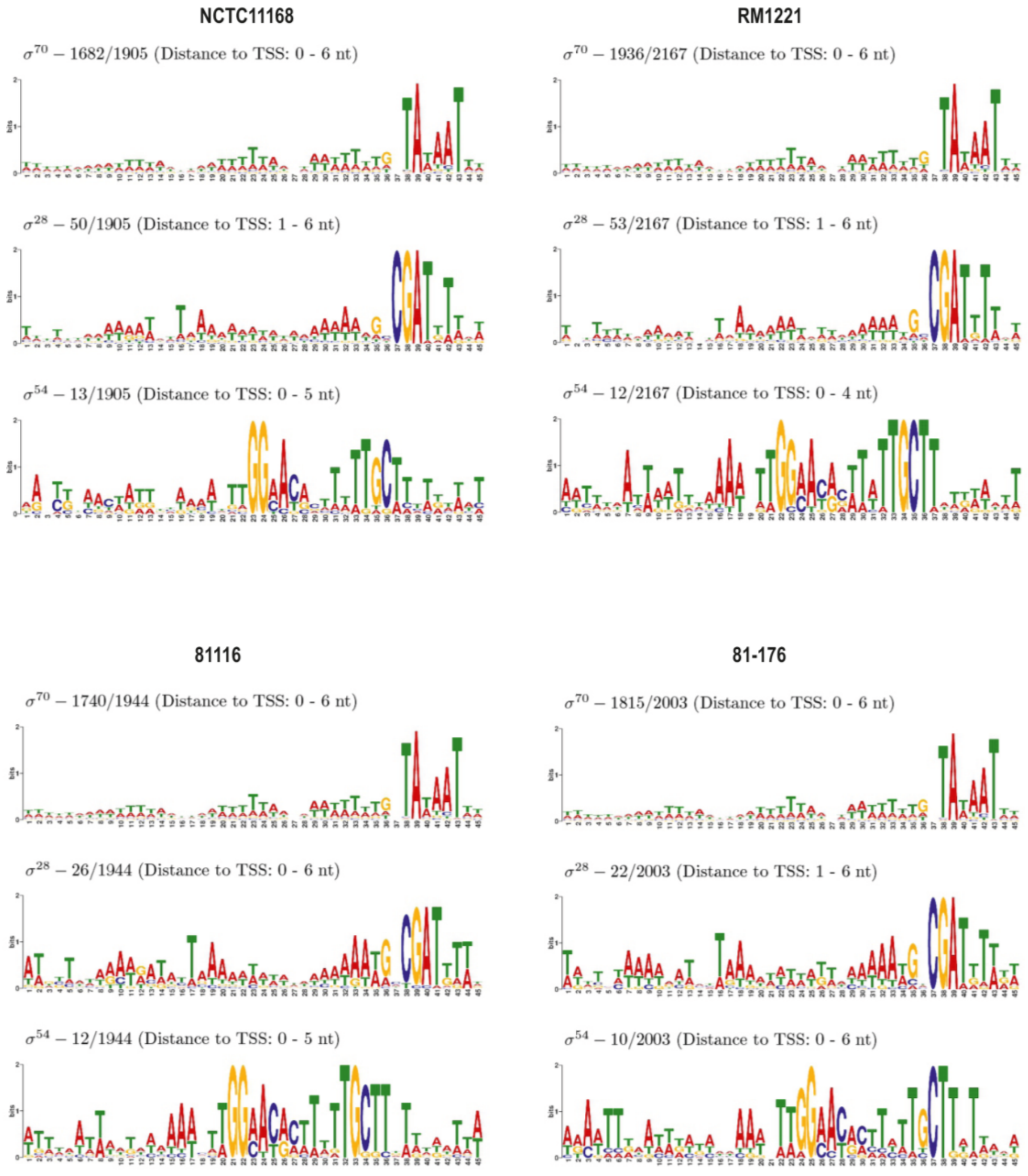


**Promoter motifs detected for TSS of the four individual strains.** Promoter motifs detected for the three sigma factors, $\sigma^{70}$, $\sigma^{28}$, and $\sigma^{54}$, in the -50 to +1 sequences of the TSS for each of the four *C. jejuni* strains. The number of occurrences for each motif is indicated. The search was performed using *MEME* and a fixed motif size of 45 nt. The range of distances to the TSS for each motif is given. The results are based on 1,905 TSS upstream sequences of *C. jejuni* NCTC11168, 2,167 of *C. jejuni* RM1221, 1,944 of *C. jejuni* 81116 and 2,003 of *C. jejuni* 81-176.

**Figure S5**

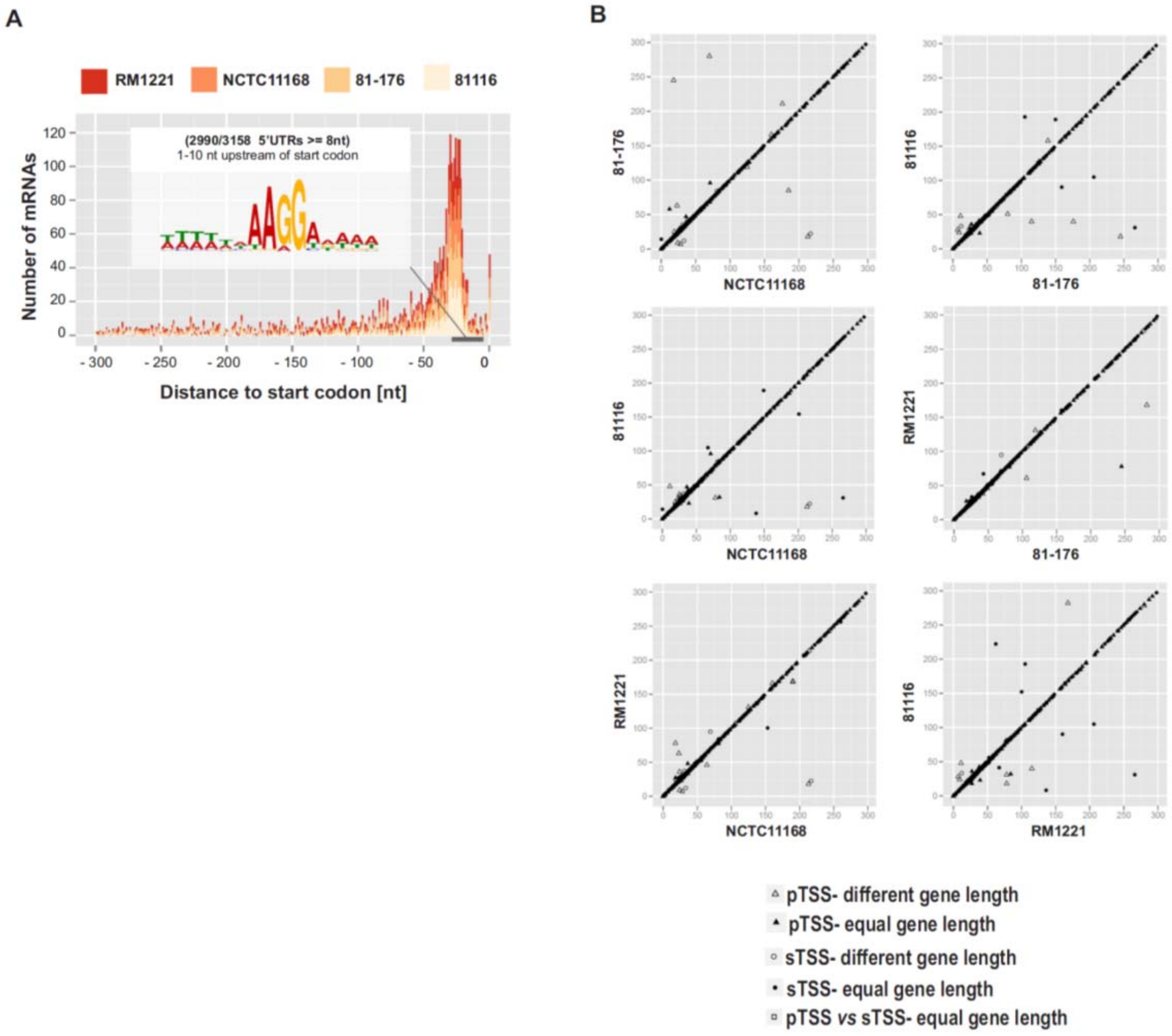


**5'UTR length distributions and comparisons. (A)** 5'UTR length distribution for 3,241 primary and secondary SuperGenome TSS (pTSS and sTSS) of mRNAs of the four *C. jejuni* strains (81116: 633 pTSS and 159 sTSS; 81-176: 628 pTSS and 168 sTSS; NCTC11168: 622 pTSS and 154 sTSS; RM1221: 698 pTSS and 179 sTSS) plotted as the frequency of the distances between primary or secondary TSS to the start codon. The inset shows the consensus ribosome binding site motif (AAGGa) that was found at a distance of 1 to 10 nt upstream of the start codon in 95 % of the 3,158 5'UTR sequences which have a minimum length of 8 nt. **(B)** For each pair of strains, the 5'UTR lengths of orthologuos genes (defined via a *BLAST* based analysis – see Supplementary Methods) of the four strains were compared pairwise and visualized as a scatter plot. The dot symbols of the plots represent the different types of comparisons of the associated TSS (primary *vs.* primary = triangle, secondary *vs.* secondary = circle, primary *vs.* secondary = square) and length comparison of the two orthologs (filled symbol = genes have the same length; empty symbol = genes have different length). If one or both genes of an ortholog pair had more than one assigned 5'UTR length, i.e. in the case of multiple promoters, the 5'UTR pairs with minimal difference were compared.

## Figure S6

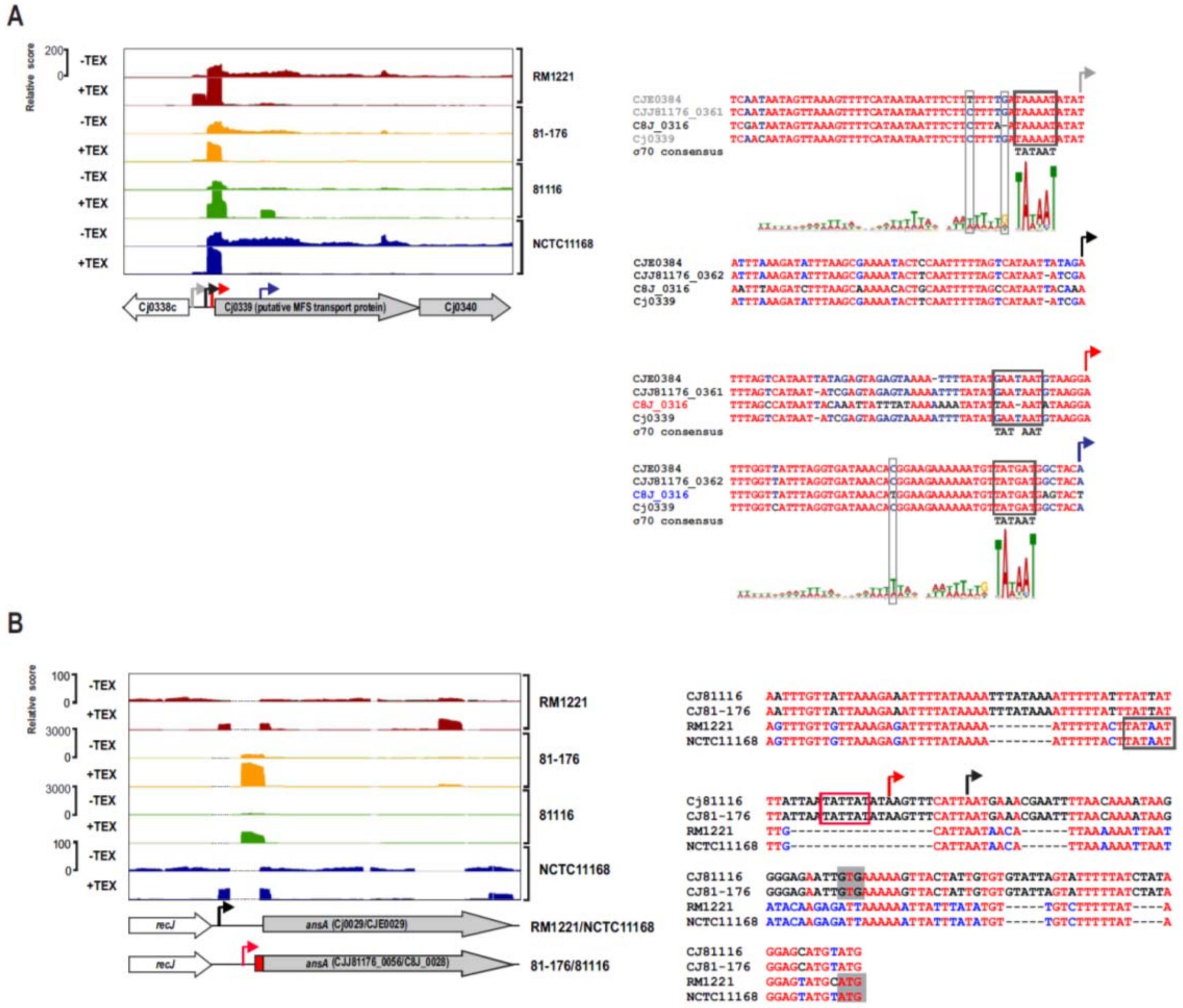


**Examples for orthologous genes with differences in 5'UTR length. (A)** *(Left)* dRNA-seq reads mapped to the Cj0339-Cj0340 operon reveal multiple TSS upstream of Cj0339. *(Right)* Alignments of the -50 to +1 regions for the TSSs shown on the left for four *C. jejuni* strains. Gene names in which the respective TSS was detected are marked in the same color as the TSS arrow. The TSS resulting in the longest 5'UTR for Cj0339 (light grey arrow) is missing in strain 81116, which has a shorter 5'UTR (red TSS) that is missing in all other strains. A primary TSS (black arrow) is detected in all four strains. Furthermore, only strain 81116 has an additional internal TSS (blue arrow) within Cj0339. **(B)** dRNA-seq reads mapped to *ansA* encoding L-asparaginase *(left)* and alignment of the *ansA* promoter and 5'UTR *(right)*. The *ansA* gene harbours a signal sequence in strain 81-176, which is marked by a red-filled box upstream of the grey-filled arrow indicating the CDS. In the alignment, the start codon of *ansA* is marked in grey and differs among strains. In strain 81116, the annotation for *ansA* is too short but can be extended to the signal sequence. Note that *ansA* is only weakly expressed in NCTC11168 and RM1221 (different scales). The dRNA-seq reads reveal two different TSS: 1) Black arrow: a TSS in RM1221 and NCTC11168 leading to a long 5'UTR and 2) Red arrow: a TSS in 81-176 and 81116 leading to a short 5'UTR due the presence of the signal sequence. The alternative TSS have -10 boxes for $\sigma^{70}$ at different positions. The dotted lines (in the genome browser snapshot) represent the gaps in the alignment of the four genomes.

**Figure S7**

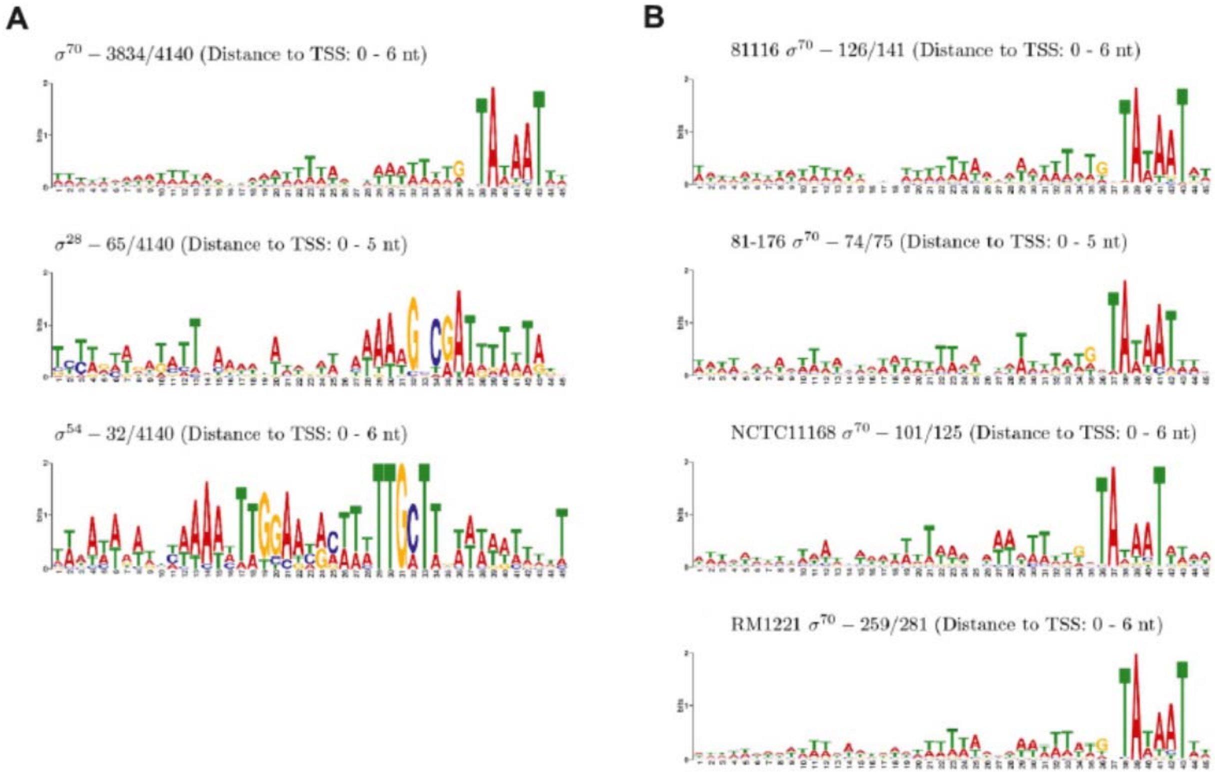


**Promoter Motifs detected by *MEME* for conserved and strain-specific TSS. (A)** Promoter motifs detected by *MEME* searches for the three sigma factors, $\sigma^{70}$, $\sigma^{28}$ and $\sigma^{54}$, which were detected in the -50 to +1 sequences upstream of conserved TSS, i.e. TSS which were detected in all four strains. The number of hits, the total number of analyzed sequences (4,140 TSS in total which corresponds to 1,035 TSS in each strain) and the range of distances of the motif to the TSS are indicated. The motif size was set to 45 nt. **(B)** $\sigma^{70}$ promoter motifs found in the -50 to +1 sequences upstream of the TSS, which were detected only in one strain. The number of sequences containing the motif and the total number of analyzed sequences as well as the distance range of the motif to the TSS are indicated. The motif size was set to 45 nt.

**Figure S8A-D**

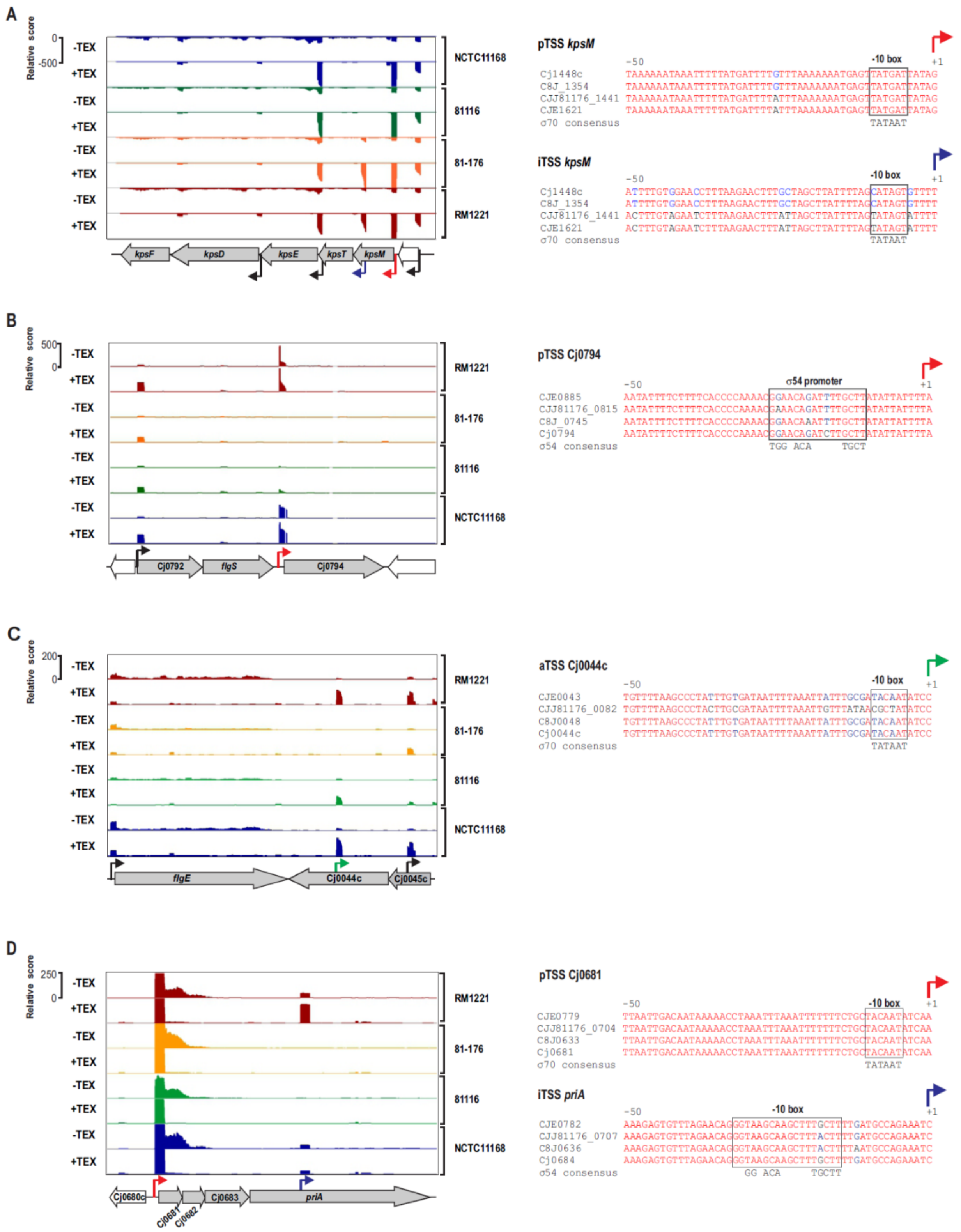
A
Relative score
0
-500
-TEX
+TEX
NCTC11168
81116
81-176
RM1221
kpsF
kpsD
kpsE
kpsT
kpsM
pTSS kpsM
-50
-10 box
+1
Cj1448c
C8J_1354
CJJ81176_1441
CJE1621
σ70 consensus
TATAAT
iTSS kpsM
B
500
0
pTSS Cj0794
σ54 promoter
CJE0885
CJJ81176_0815
C8J_0745
Cj0794
σ54 consensus
TGG ACA
TGCT
Cj0792
flgS
Cj0794
C
200
aTSS Cj0044c
CJE0043
CJJ81176_0082
C8J0048
Cj0044c
flgE
Cj0044c
Cj0045c
D
250
pTSS Cj0681
CJE0779
CJJ81176_0704
C8J0633
Cj0681
iTSS priA
CJE0782
CJJ81176_0707
C8J0636
Cj0684
GG ACA
TGCTT
Cj0680c
Cj0681
Cj0682
Cj0683
priA

**Figure S8E-H**

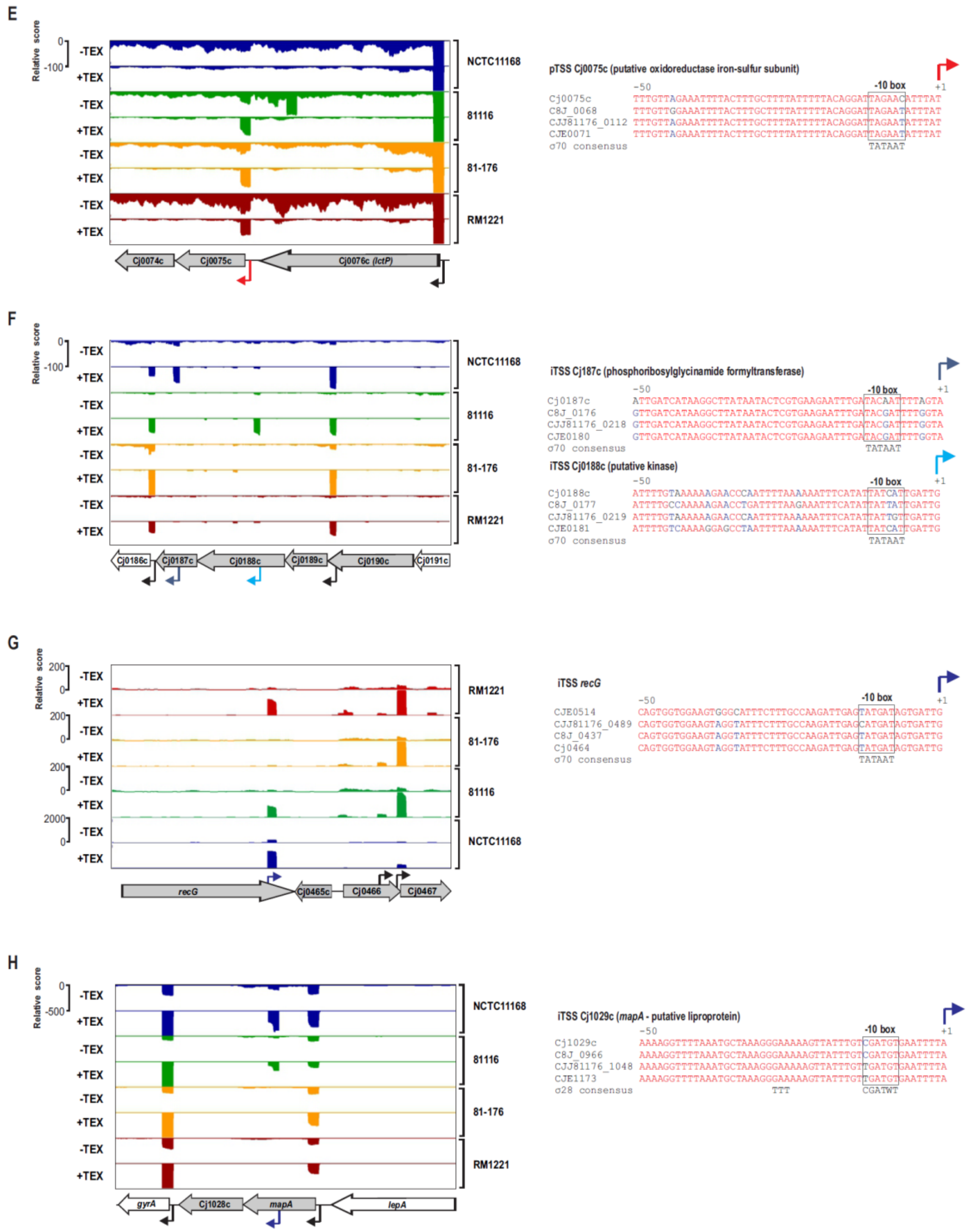
E
Relative score
0
-100
-TEX
+TEX
NCTC11168
81116
81-176
RM1221
Cj0074c
Cj0075c
Cj0076c (lctP)
pTSS Cj0075c (putative oxidoreductase iron-sulfur subunit)
-50
-10 box
+1
Cj0075c
C8J_0068
CJJ81176_0112
CJE0071
σ70 consensus
TATAAT
F
Relative score
0
-100
-TEX
+TEX
NCTC11168
81116
81-176
RM1221
Cj0186c
Cj0187c
Cj0188c
Cj0189c
Cj0190c
Cj0191c
iTSS Cj187c (phosphoribosylglycinamide formyltransferase)
-50
-10 box
+1
Cj0187c
C8J_0176
CJJ81176_0218
CJE0180
σ70 consensus
TATAAT
iTSS Cj0188c (putative kinase)
-50
-10 box
+1
Cj0188c
C8J_0177
CJJ81176_0219
CJE0181
σ70 consensus
TATAAT
G
Relative score
200
0
2000
-TEX
+TEX
RM1221
81-176
81116
NCTC11168
recG
Cj0465c
Cj0466
Cj0467
iTSS recG
-50
-10 box
+1
CJE0514
CJJ81176_0489
C8J_0437
Cj0464
σ70 consensus
TATAAT
H
Relative score
0
-500
-TEX
+TEX
NCTC11168
81116
81-176
RM1221
gyrA
Cj1028c
mapA
lepA
iTSS Cj1029c (mapA - putative liproprotein)
-50
-10 box
+1
Cj1029c
C8J_0966
CJJ81176_1048
CJE1173
σ28 consensus
TTT
CGATWT

**Figure S8I-L**

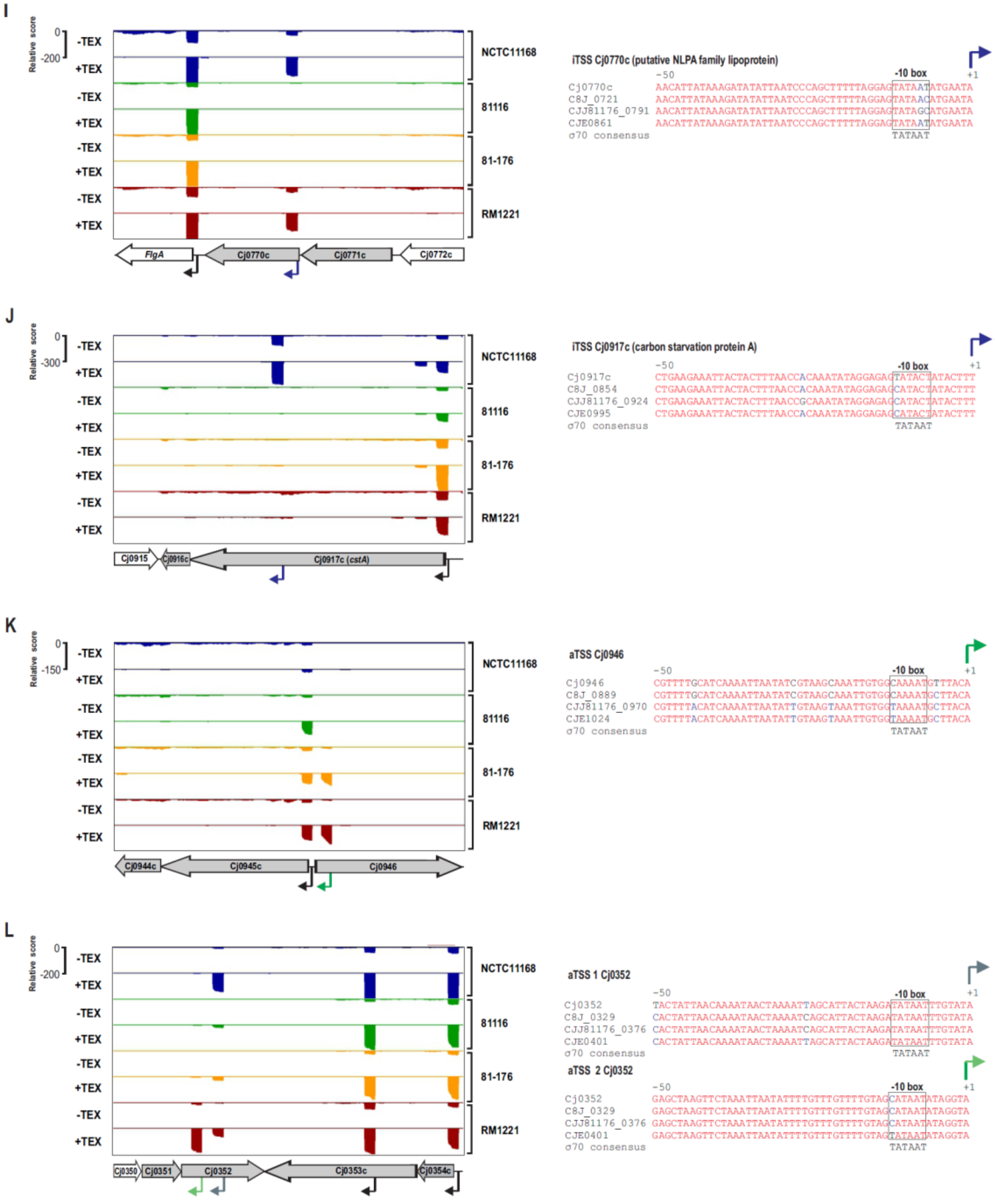
I
Relative score
0
-200
-TEX
+TEX
NCTC11168
81116
81-176
RM1221
FlgA
Cj0770c
Cj0771c
Cj0772c
iTSS Cj0770c (putative NLPA family lipoprotein)
-50
-10 box
+1
Cj0770c AACATTATAAAGATATATTAATCCCAGCTTTTTAGGAGTATAATATGAATA
C8J_0721 AACATTATAAAGATATATTAATCCCAGCTTTTTAGGAGTATAACATGAATA
CJJ81176_0791 AACATTATAAAGATATATTAATCCCAGCTTTTTAGGAGTATAGCATGAATA
CJE0861 AACATTATAAAGATATATTAATCCCAGCTTTTTAGGAGTATAATATGAATA
σ70 consensus TATAAT
J
Relative score
0
-300
Cj0915
Cj0916c
Cj0917c (cstA)
iTSS Cj0917c (carbon starvation protein A)
Cj0917c CTGAAGAAATTACTACTTTAACCACAAATATAGGAGAGTATACTATACTTT
C8J_0854 CTGAAGAAATTACTACTTTAACCACAAATATAGGAGAGCATACTATACTTT
CJJ81176_0924 CTGAAGAAATTACTACTTTAACCGCAAATATAGGAGAGCATACTATACTTT
CJE0995 CTGAAGAAATTACTACTTTAACCACAAATATAGGAGAGCATACTATACTTT
σ70 consensus TATAAT
K
Relative score
0
-150
Cj0944c
Cj0945c
Cj0946
aTSS Cj0946
Cj0946 CGTTTTGCATCAAAATTAATATCGTAAGCAAATTGTGGCAAAATGTTTACA
C8J_0889 CGTTTTGCATCAAAATTAATATCGTAAGCAAATTGTGGCAAAATGCTTACA
CJJ81176_0970 CGTTTTACATCAAAATTAATATTGTAAGTAAATTGTGGTAAAATGCTTACA
CJE1024 CGTTTTACATCAAAATTAATATTGTAAGTAAATTGTGGTAAAATGCTTACA
σ70 consensus TATAAT
L
Relative score
0
-200
Cj0350
Cj0351
Cj0352
Cj0353c
Cj0354c
aTSS 1 Cj0352
Cj0352 TACTATTAACAAAATAACTAAAATTAGCATTACTAAGATATAATTTGTATA
C8J_0329 CACTATTAACAAAATAACTAAAATCAGCATTACTAAGATATAATTTGTATA
CJJ81176_0376 CACTATTAACAAAATAACTAAAATCAGCATTACTAAGATATAATTTGTATA
CJE0401 CACTATTAACAAAATAACTAAAATTAGCATTACTAAGATATAATTTGTATA
σ70 consensus TATAAT
aTSS 2 Cj0352
Cj0352 GAGCTAAGTTCTAAATTAATATTTTGTTTGTTTTGTAGCATAATATAGGTA
C8J_0329 GAGCTAAGTTCTAAATTAATATTTTGTTTGTTTTGTAGCATAATATAGGTA
CJJ81176_0376 GAGCTAAGTTCTAAATTAATATTTTGTTTGTTTTGTAGCATAATATAGGTA
CJE0401 GAGCTAAGTTCTAAATTAATATTTTGTTTGTTTTGTAGTATAATATAGGTA
σ70 consensus TATAAT

**dRNA-seq data and promoter alignments for genes with differentially expressed TSS among four *C. jejuni* strains***.* *(Left)* dRNA-seq reads mapped to different regions of the SuperGenome with differential TSS usage among strains. *(Right)* Alignments of the -50 to +1 regions upstream of the TSS shown in the dRNA-seq data on the left for the four different *C. jejuni* strains. **(A)** dRNA-seq data of the four strains mapped to the capsule export genes *kpsMTEDF*. Black arrows indicate TSS that were detected within this region based on enrichment in the TEX+ libraries of all four strains. The primary TSS of *kpsM* (red arrow) is enriched in all four strains. In contrast, the internal TSS (blue arrow) within *kpsM* is present in only two strains (81-176 and RM1221), probably due to point mutations in the respective promoter region of NCTC11168 and 81116 as visible in the alignment on the right**.** **(B)** dRNA-seq reads mapped to Cj0794, which encodes a hypothetical protein, indicate differential expression from its primary TSS. The promoter region with the non-detected TSS in strain 81-176 shows mutations within a $\sigma^{54}$ consensus sequence. **(C)** A *cis*-encoded antisense RNA to Cj0044c is differentially expressed among the strains and has a disrupted $\sigma^{70}$ -10 box in strain 81-176. **(D)** Differential expression of an internal TSS within *priA* (blue arrow) coincides with mutations in a $\sigma^{54}$ box in strains 81-176 and 81116. **(E)** Differential expression of the pTSS of Cj0075c (red arrow) due to a T to C exchange at the conserved last "T" of the $\sigma^{70}$ -10 box in strain NCTC11168. **(F)** Two internal TSS (blue arrows) were detected in Cj0187c and Cj0188c. These iTSS (Cj0187c, dark blue; Cj0188c, light blue) are only transcribed in strains NCTC11168 or 81116. **(G)** Differential expression of an iTSS (blue arrow) at the 3' end of *recG* which shows a T to C exchange at the conserved first "T" residue of the $\sigma^{70}$ -10 box in 81-176. Note that the scale for NCTC11168 has been changed to 0-2,000 due to high levels of this internal transcript compared to 81116 and RM1221. **(H)** Differential expression of an iTSS (blue arrow) in *mapA* is probably due to disruption of a putative $\sigma^{28}$ box in 81-176 and RM1221. **(I)** Differential expression of an iTSS (blue arrow) at the 5' end of Cj0770c coincides with disruption of the $\sigma^{70}$ -10 box in strains 81116 and 81-176. **(J)** An iTSS (blue arrow) within *cstA* is only transcribed in NCTC11168 which carries an intact $\sigma^{70}$ -10 box upstream. **(K)** A *cis*-encoded antisense RNA (green arrow) at the 5' end of Cj0946 is transcribed only in strains 81-176 and RM1221. **(L)** Two *cis*-encoded antisense RNAs to Cj0352 with differential expression among strains*.* The aTSS1 (dark green arrow) along with its promoter is conserved in all strains. In contrast, aTSS2 (green arrow) is only transcribed in strain RM1221, whereas all other strains have a point mutation in the -10 box.

**Figure S9**

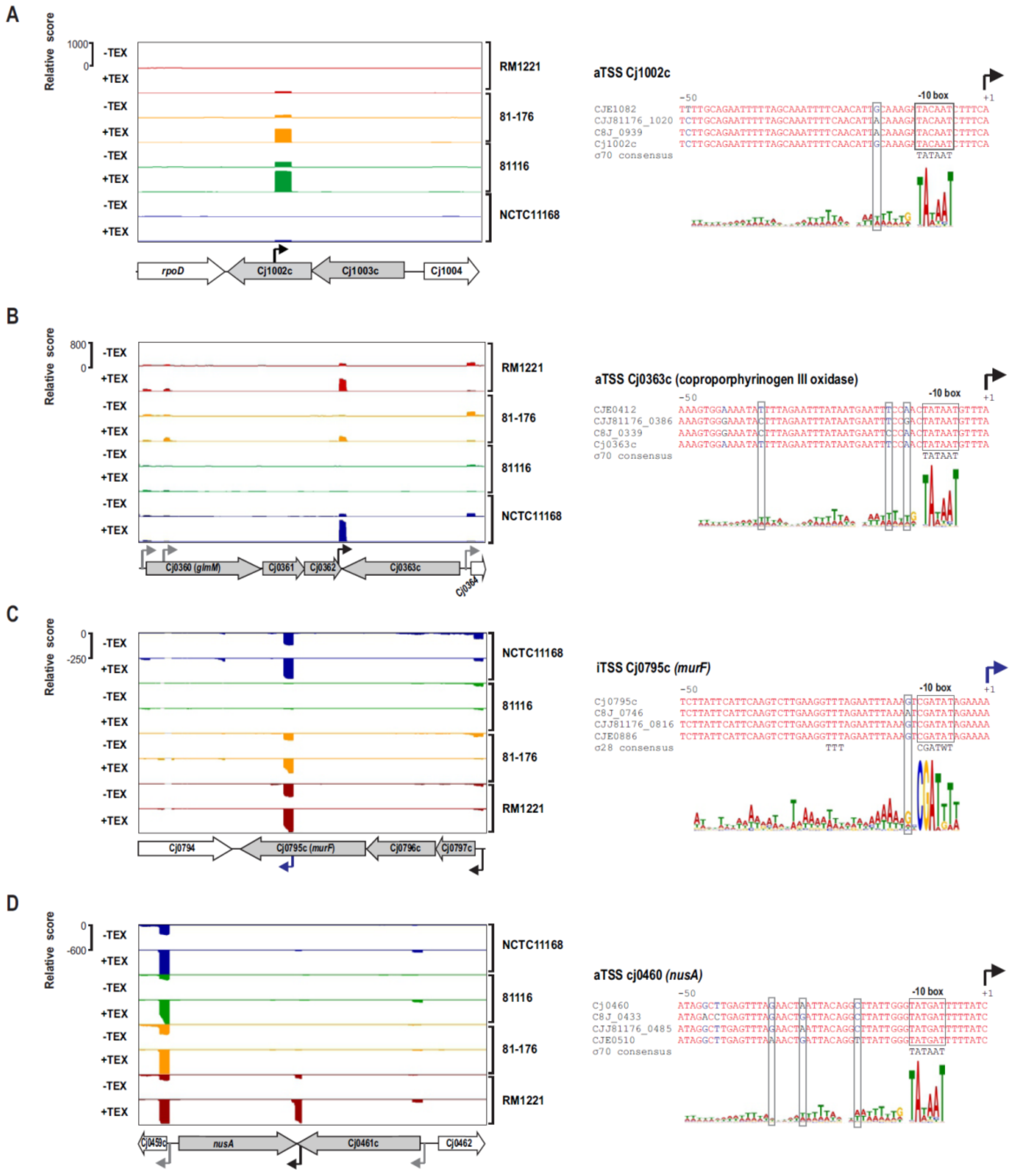


**Differentially expressed TSS with SNPs in the periodic A/T-rich pattern or in the extended -10 promoter box.** dRNA-seq reads mapped to different genomic loci. **(A)** *(Left)* dRNA-seq reads mapped antisense to the Cj1003c-Cj1002c operon reveal a novel conserved *cis*-encoded antisense RNA to Cj1002c. (*Right*) The promoter corresponding to this asTSS has a conserved $\sigma^{70}$ -10 box but shows an A to G exchange in the A/T-rich cyclic pattern upstream of the -10 box in strains RM1221 and NCTC11168, which apparently leads to loss of transcription from this aTSS in these strains. **(B)** A differentially expressed *cis*-encoded antisense RNA (black arrow) to

Cj0363c (coproporphyrinogen III oxidase) shows mutations in the A/T-rich upstream region in strain 81116. **(C)** A differentially transcribed iTSS (blue arrow) in Cj0795c *(murF)* is not transcribed in strain 81116 probably due to a mutation at a conserved G residue two nucleotides upstream of a $\sigma^{28}$ box. **(D)** A differentially expressed *cis*-encoded antisense RNA (black arrow) to Cj0460 *(nusA)* is only expressed in strain RM1221 and carries point mutations in the A/T-rich upstream region in the three other strains.

**Figure S10**

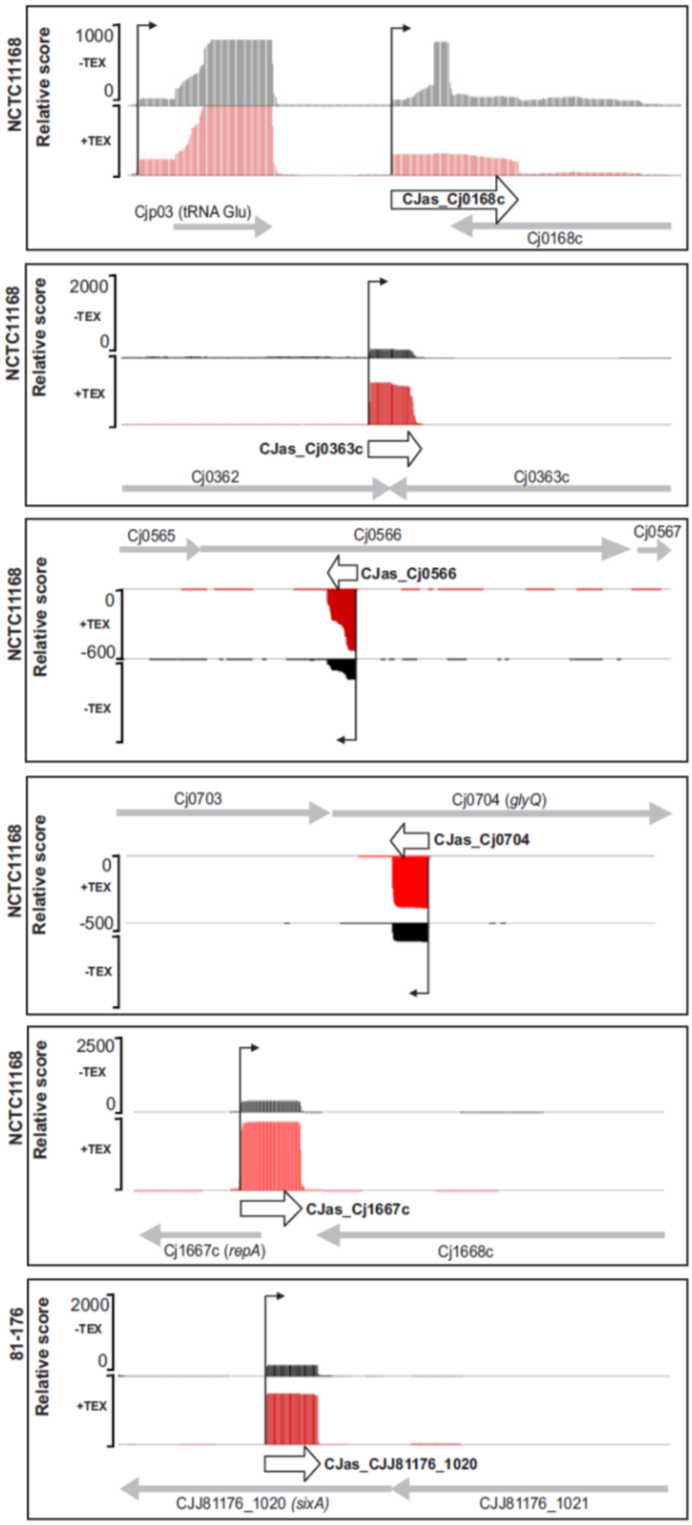


**Examples for genes with *cis*-encoded antisense RNAs.** Sequenced reads of cDNA libraries derived from -/+TEX-treated total RNA mapped to several antisense RNA loci in the chromosome of different strains of *C. jejuni.* Strain names are indicated on the left of the screen shots. The antisense transcripts were named CJas_"X" according to the name of gene "X" encoded on the opposite strand. The selected examples had at least 100 cDNA reads in the TEX- library.

## Figure S11

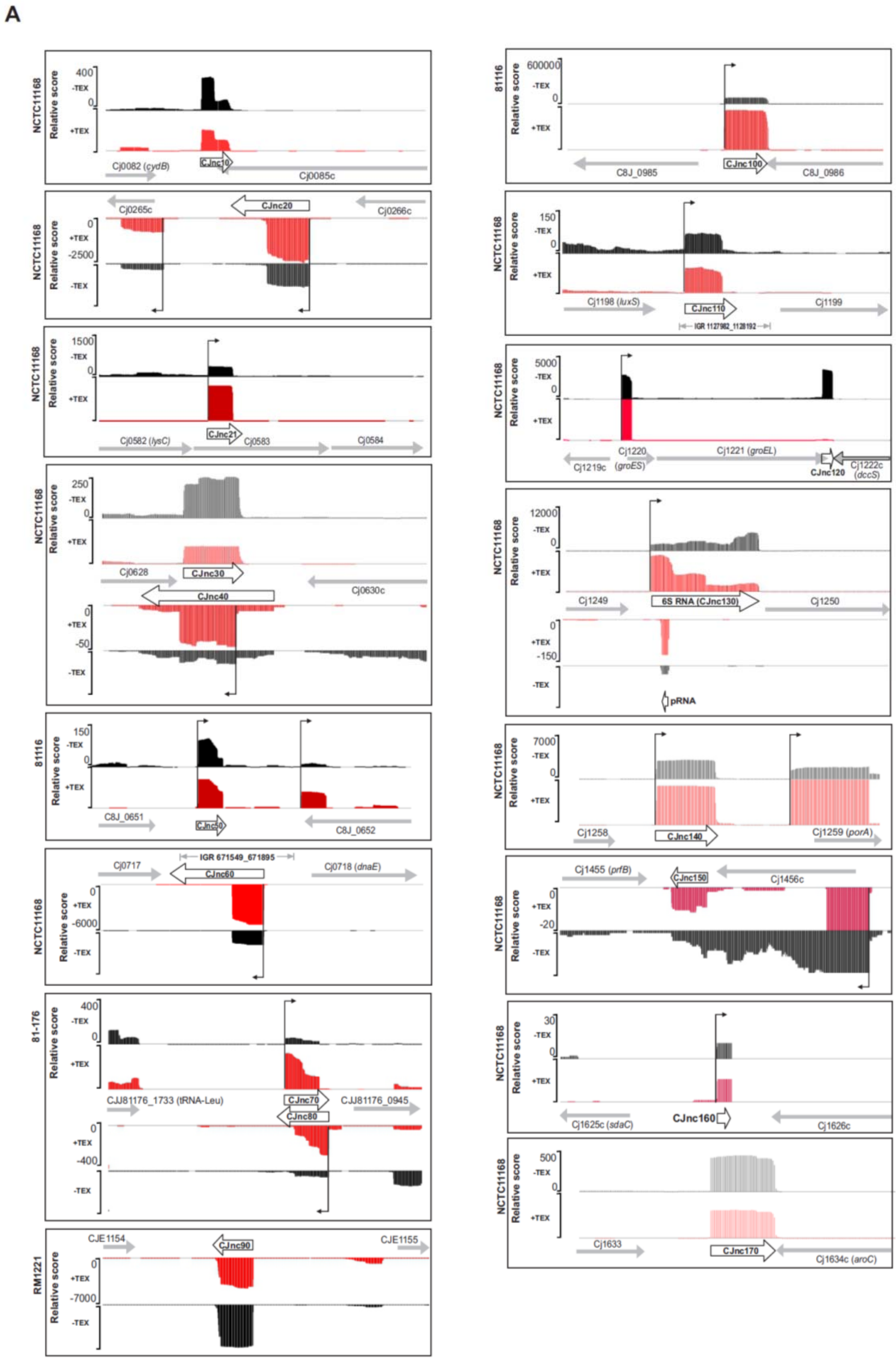
A
NCTC11168
Relative score
400
-TEX
0
+TEX
Cj0082 (cydB)
CJnc10
Cj0085c
Cj0265c
CJnc20
Cj0266c
-2500
1500
Cj0582 (lysC)
CJnc21
Cj0583
Cj0584
250
Cj0628
CJnc30
Cj0630c
CJnc40
-50
81116
150
C8J_0651
CJnc50
C8J_0652
IGR 671549_671895
Cj0717
CJnc60
Cj0718 (dnaE)
-6000
81-176
CJJ81176_1733 (tRNA-Leu)
CJnc70
CJnc80
CJJ81176_0945
-400
RM1221
CJE1154
CJnc90
CJE1155
-7000
600000
C8J_0985
CJnc100
C8J_0986
Cj1198 (luxS)
CJnc110
Cj1199
IGR 1127982_1128192
5000
Cj1219c
Cj1220 (groES)
Cj1221 (groEL)
CJnc120
Cj1222c (dccS)
12000
Cj1249
6S RNA (CJnc130)
Cj1250
-150
pRNA
7000
Cj1258
CJnc140
Cj1259 (porA)
Cj1455 (prfB)
CJnc150
Cj1456c
-20
30
Cj1625c (sdaC)
CJnc160
Cj1626c
500
Cj1633
CJnc170
Cj1634c (aroC)

## Figure S11A (continued)-B

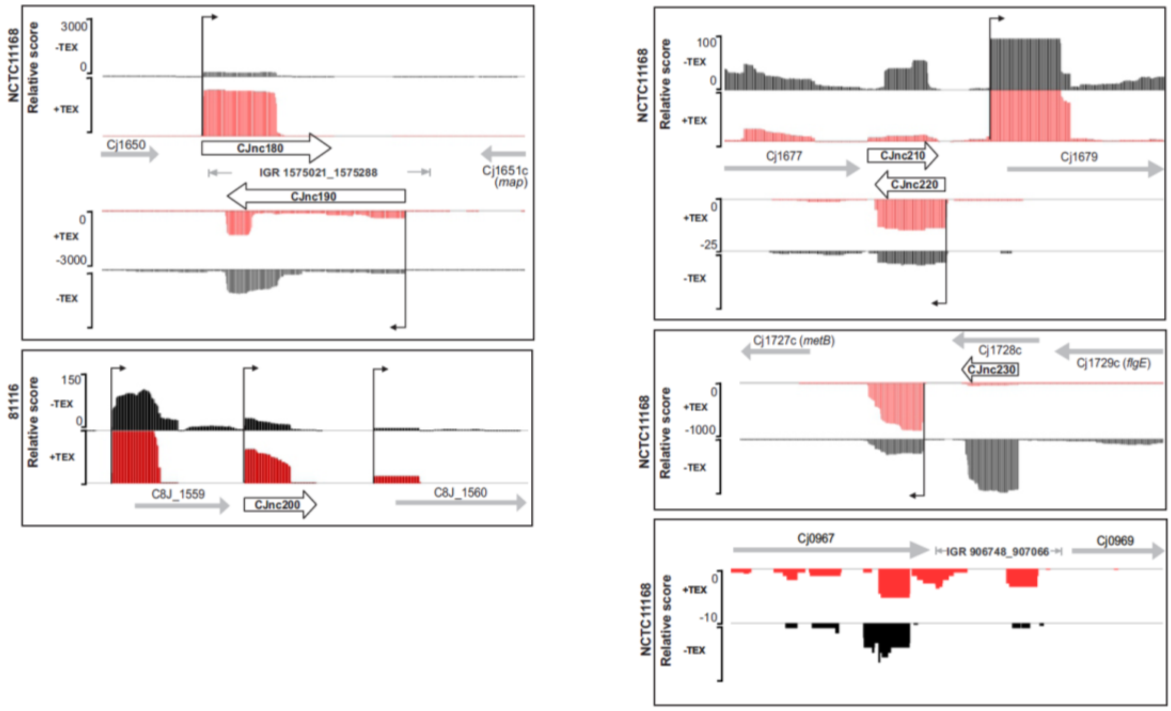


**B**

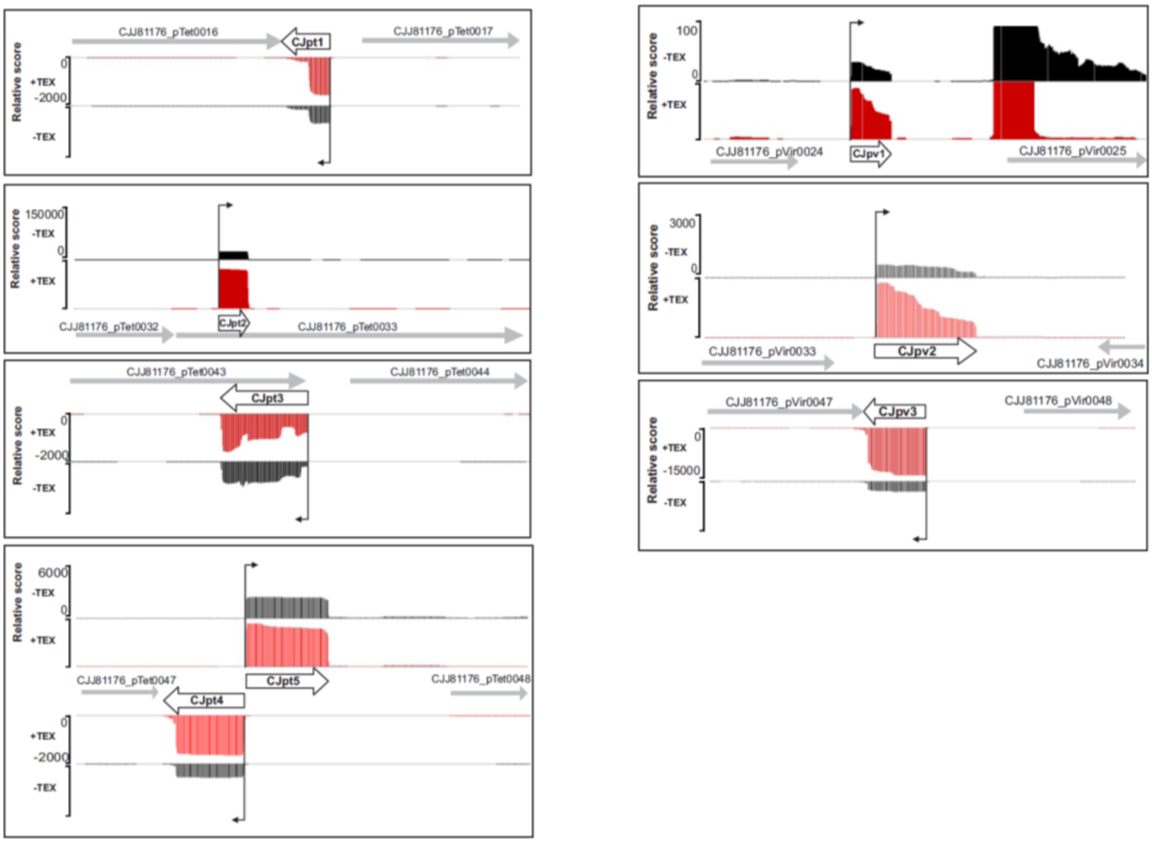

**dRNA-seq reveals several sRNA candidates in *C. jejuni.*** Sequenced reads of cDNA libraries derived from -/+TEX-treated total RNA mapped to candidate sRNA loci. Regarding nomenclature, sRNA candidates were termed “CJncXX” or “CJncXXX” and numbered in steps of ten according to the genome position of the TSS of putative small RNA. Exact coordinates are listed in Table S11. Steps of ten were chosen to leave the option to accommodate additional sRNAs. TSS with enrichment in the TEX+ library are indicated by black arrows. **(A)** Screen-shots of cDNA reads mapped to sRNAs loci in the chromosomes of different *C. jejuni* strains*.* The respective strain name is indicated on the left of each screen shot. Note that IGR regions with potential sRNAs candidates indentified by Chaudhuri *et al.* [16] which overlap with sRNA candidates identified in this study or for which we detected cDNA reads are also indicated in the screenshots. Most sRNAs are transcribed from their own TSS. However, for example a sRNA candidate, CJnc120, downstream of *groEL* seems to be generated by processing from the 3’end of the *groEL* mRNA since it is enriched in the TEX- library. Moreover, also CJnc230 seems to be generated by processing. **(B)** Screen shots of dRNA-seq reads mapped to plasmid encoded sRNA loci in the pTet and the pVir plasmids from 81-176. TSS of sRNAs are indicated by black arrows. Exact coordinates are listed in Table S12.

**Figure S12**

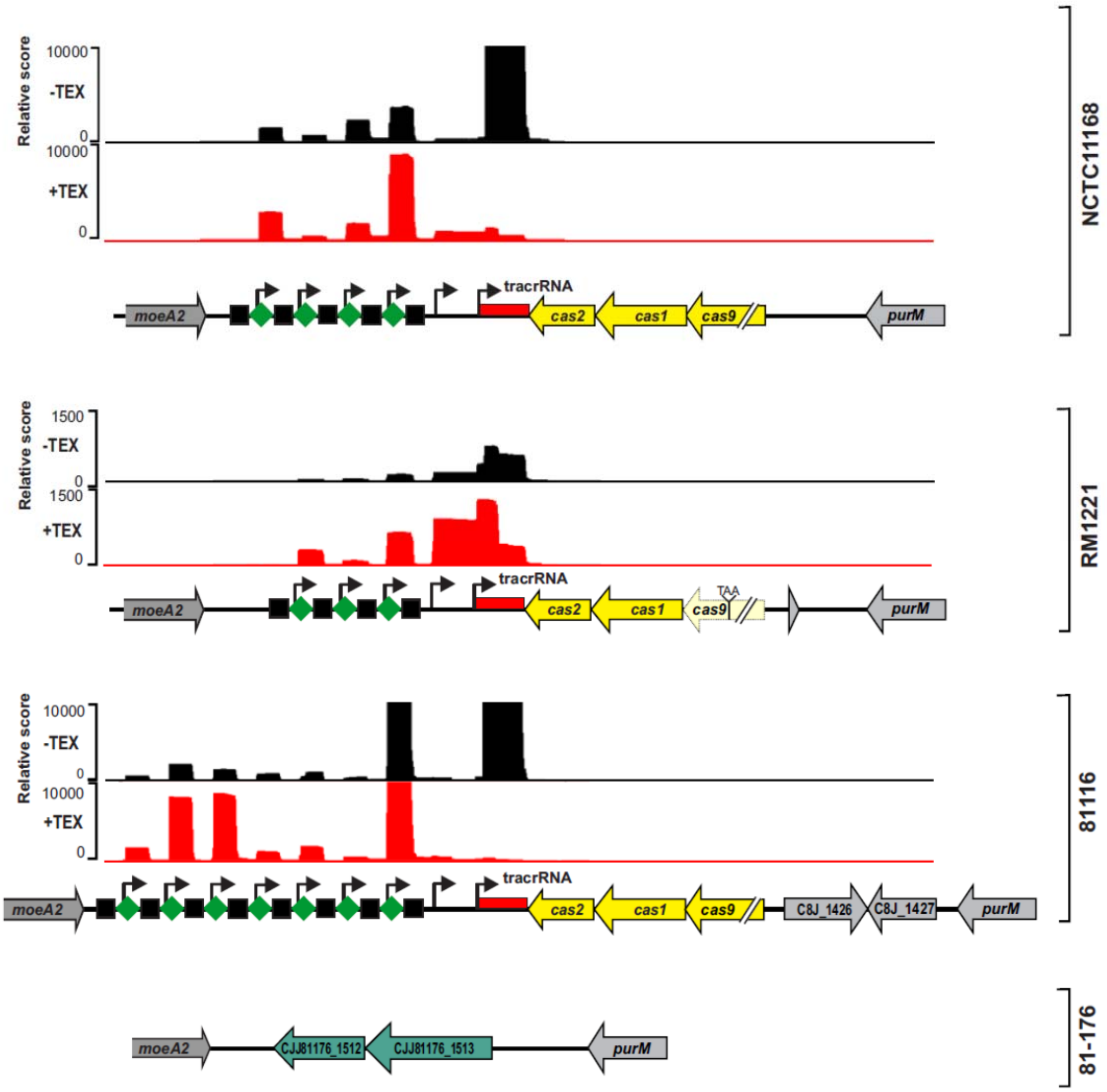


**Transcription and genomic organization of type-II C CRISPR/*cas* loci in *C. jejuni*.** Sequenced cDNAs reads derived from -/+TEX-treated total RNA that were mapped to the CRISPR loci in *C. jejuni* NCTC11168, RM1221, and 81116. Black squares indicate the CRISPR repeats, green diamonds the CRISPR spacers, and the red rectangle the tracrRNA gene, respectively. Enrichment of the 5'ends of mature crRNAs in the TEX+ treated cDNA library indicates the presence of a TSS within each spacer in the CRISPR locus. The processed TracrRNA is enriched in the untreated cDNA library, whereas enrichment of the 5'-end of the TracrRNA precursor shows the presence of a promoter upstream of the tracrRNA gene. Locations of the crRNA promoters and the tracrRNA promoter are indicated by black arrows. The *cas* genes (*cas9-cas1-cas2*) are encoded in opposite direction to tracrRNA and the CRISPR spacer-repeat array. Note that the CRISPR/*cas* locus in *C. jejuni* 81-176 is replaced by two A/T-rich genes encoding for hypothetical proteins.

**Figure S13**

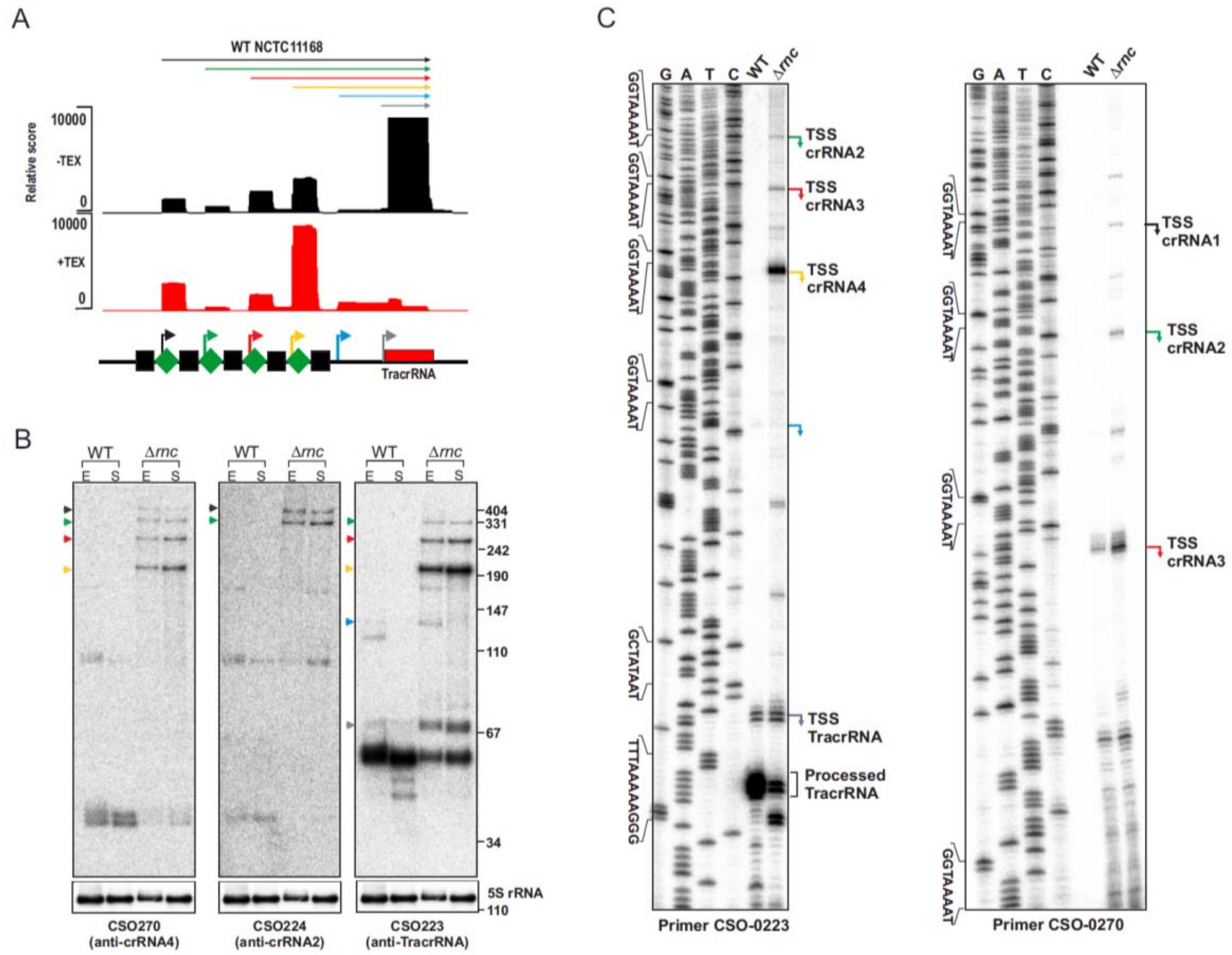


**Deletion of RNase III leads to accumulation of longer crRNAs. (A)** Sequenced reads of cDNA libraries derived from -/+TEX-treated total RNA mapped to the CRISPR loci in *C. jejuni* NCTC11168. Arrows indicate transcripts initiating from the different TSS found in the locus. **(B)** Northern blot analysis of TracrRNA and crRNAs in NCTC11168 wild-type (WT) and *Δrnc*. Total RNA (10 µg per lane) extracted from NCTC11168 WT and *Δrnc* at mid-exponential (E) and stationary (S) phase was analyzed on a Northern blot using labeled DNA probes complementary to crRNA4, crRNA2 and TracrRNA in NCTC11168. **(C)** Primer extension analysis to determine the 5' ends of different transcripts in the CRISPR locus in NCTC11168 WT and *Δrnc* strains. Labeled DNA probes, CSO-0223 and CSO-0270, were incubated with 5 µg of mid-exponential phase RNA from NCTC11168 WT and *Δrnc* in the RT reaction. Lanes G, A, T, C represent sequencing reactions carried out on an extended CRISPR locus sequence of NCTC11168 amplified using oligos CSO-0223 and CSO-0270. The arrows indicate the identified TSS among other processed fragments. The $\sigma^{70}$ -10 boxes of the TSS observed along with the TracrRNA processing site are marked on the left of the gel.

**Figure S14**

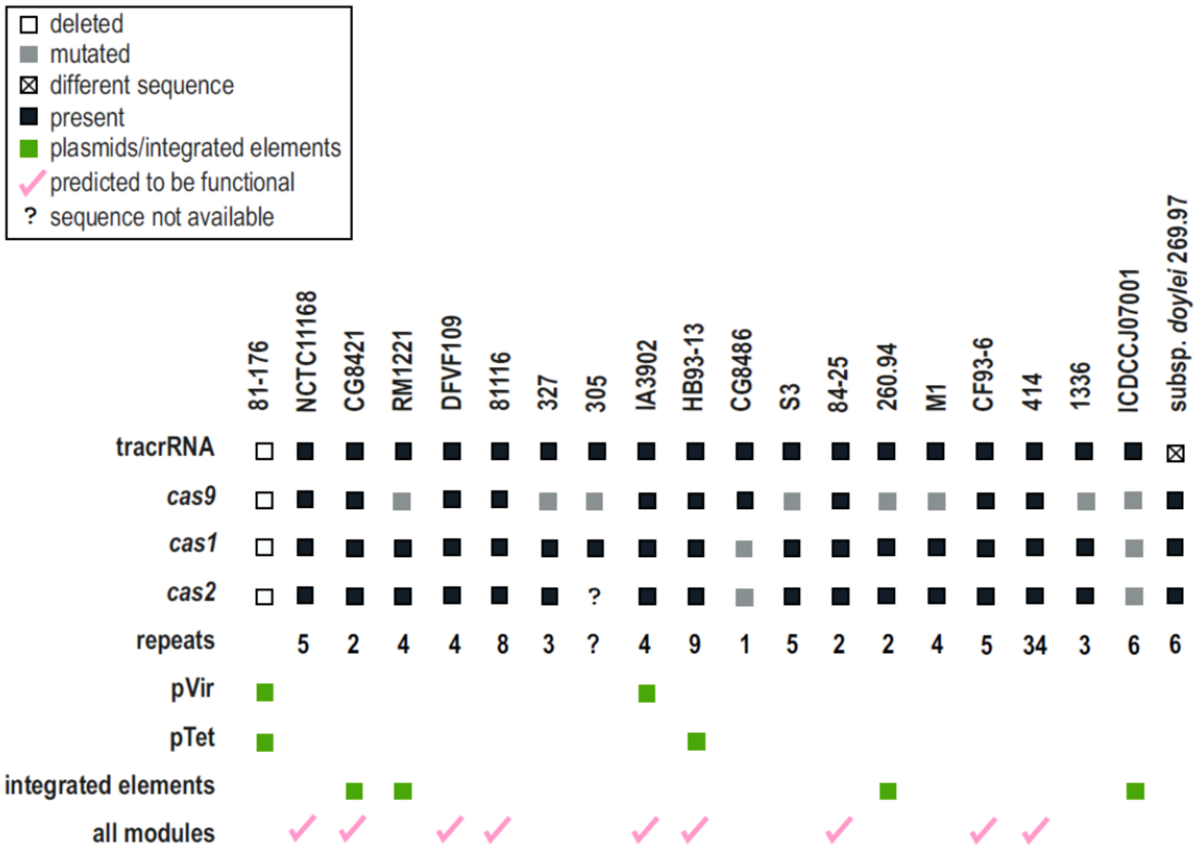


**Correlation analysis of intact CRISPR loci and the presence/absence of plasmids or integrated elements in diverse *C. jejuni* clinical isolates.** Only nine out of twenty *C. jejuni* strains with available genome sequence are predicted to have functional CRISPR/*cas* modules and TracrRNA sequences based on conservation analysis. Interestingly, in the majority of strains with predicted functional CRISPR/*cas* and tracrRNA genes no extra-chromosomal elements can be found. However, for three strains, *C. jejuni* CG8421, IA3902, and HB93-13, that seem to have functional CRISPR/*cas* loci, also plasmids and integrated elements are present. Intact TracrRNA homologs can be predicted for nine genomes that harbor only deleted or mutated CRISPR/*cas* loci. This indicates that there is no correlation between the presence of a conserved TracrRNA and a functional CRISPR/*cas* locus in those species. For strain *C. jejuni* subsp. *doylei,* the TracrRNA sequence is not conserved, indicating that the CRISPR/cas system might not be active in this strain.

**Figure S15**

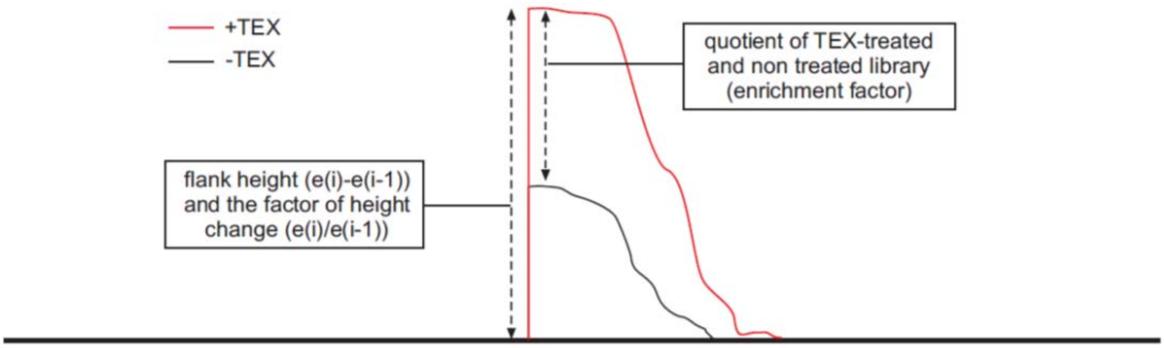


**Schematic representation of basic TSS detection criteria based on dRNA-seq data**. The dRNA-seq expression graphs from the exonuclease (TEX) treated (red) and untreated (black) cDNA libraries are the basis for the TSS detection procedure. The most important parameters that are considered during the process are the flank height at position i of the potential TSS (e(i)-e(i-1)) and the factor of height change at that position (e(i)/e(i-1)), where e(i) is the expression value at position i. Additionally, the enrichment factor at the same position is taken into account (i.e. the quotient of the expression values from the treated and untreated library).

## Legends Supplementary Tables S3-5

**Table S3. SuperGenome TSS table.**

This table is provided as a separate Excel sheet.

The table contains information on positions and assigned classes of all annotated TSS. It lists all TSS that were detected in the SuperGenome (column "detected" = 1). In case a TSS was not detected in a certain strain the value of detected is "0". If a TSS was mapped in more than one genome via the SuperGenome, there is one row for each genome the TSS was mapped to. Also, if the TSS is assigned to more than one class, there is one row for each class assignment and each associated gene. The table contains the following columns:

*SuperPos*: The position of the TSS in the SuperGenome. If a TSS is detected in more than one strain, the position in the SuperGenome is always the same but the position in the individual genomes differs.
*SuperStrand*: The strand of the TSS in the SuperGenome.
*mapCount*: The number of genomes in which this TSS could be mapped via the SuperGenome.
*detCount*: The number of genomes in which this TSS was detected in the RNAseq data.
*Genome*: The identifier of the genome to which the rest of the line relates.
*detected*: Contains a '1' if the TSS was detected in this genome.
*enriched*: Contains a '1' if the TSS is enriched in this genome.
*stepHeight*: The expression height change at the position of the TSS. This relates to the number of reads starting at this position.
*stepFactor*: The factor of height change at the position of the TSS.
*enrichmentFactor*: The enrichment factor at the position of the TSS.
*classCount*: The number of classes to which this TSS was assigned.
*Pos*: The position of the TSS in the respective genome.
*Strand*: The strand of the TSS in the respective genome.
*Locus_tag*: The locus tag of the gene to which the classification relates.
*Product*: The product description of this gene.
*UTRlength*: The length of the untranslated region between the TSS and the respective gene (nt). (Only applies to 'primary' and 'secondary' TSS.)
*GeneLength*: The length of the gene (nt).
*Primary*: Contains a '1' if the TSS was classified as 'primary' with respect to the gene stated in 'locusTag'.
*Secondary*: Contains a '1' if the TSS was classified as 'secondary' with respect to the gene stated in 'locusTag'.
*Internal*: Contains a '1' if the TSS was classified as 'internal' with respect to the gene stated in 'locusTag'.
*Antisense*: Contains a '1' if the TSS was classified as 'antisense' with respect to the gene stated in 'locusTag'.
*Automated*: Contains a '1' if the TSS was detected automatically.
*Manual*: Contains a '1' if the TSS was annotated manually.
*Putative sRNA*: Contains a '1' if the TSS might be related to a novel sRNA.
*Putative asRNA*: Contains a '1' if the TSS might be related to an asRNA.
*Sequence -50 nt upstream + TSS (51nt)*: Contains the base of the TSS and the 50 nucleotides upstream of the TSS.

**Table S4. TSS detected in the individual *C. jejuni* strains.**

This table is provided as a separate Excel sheet and lists all TSS detected in the individual *C. jejuni* strains NCTC11168, RM1221, 81116, and 81-176 in separate Excel sheets. It contains the following columns:

*Pos*: The position of the TSS in the respective genome.
*Strand*: The strand of the TSS in the respective genome.
*detected*: Contains a '1' if the TSS was detected in this genome.
*enriched*: Contains a '1' if the TSS is enriched in this genome.
*stepHeight*: The expression height change at the position of the TSS. This relates to the number of reads starting at this position.
*stepFactor*: The factor of height change at the position of the TSS.
*enrichmentFactor*: The enrichment factor at the position of the TSS.
*classCount*: The number of classes to which this TSS was assigned.
*Locus_tag*: The locus tag of the gene to which the classification relates.
*Product*: The product description of this gene.
*UTRlength*: The length of the untranslated region between the TSS and the respective gene (nt). (Only applies to 'primary' and 'secondary' TSS.)
*GeneLength*: The length of the gene (nt).
*Primary*: Contains a '1' if the TSS was classified as 'primary' with respect to the gene stated in 'locusTag'.
*Secondary*: Contains a '1' if the TSS was classified as 'secondary' with respect to the gene stated in 'locusTag'.
*Internal*: Contains a '1' if the TSS was classified as 'internal' with respect to the gene stated in 'locusTag'.
*Antisense*: Contains a '1' if the TSS was classified as 'antisense' with respect to the gene stated in 'locusTag'.
*Automated*: Contains a '1' if the TSS was detected automatically.
*Manual*: Contains a '1' if the TSS was annotated manually.
*Putative sRNA*: Contains a '1' if the TSS might be related to a novel sRNA.
*Putative asRNA*: Contains a '1' if the TSS might be related to an asRNA.
*Sequence -50 nt upstream + TSS (51nt)*: Contains the base of the TSS and the 50 nucleotides upstream of the TSS.

**Table S5. TSS detected on the pVir and pTet plasmids of *C. jejuni* 81-176 .**

This table is provided as a separate Excel sheet.

This table contains information on positions and class assignments of all TSS detected for the two plasmids of *C. jejuni* 81-176. We did not classify these TSS as being enriched or not. Instead the enrichment factor is provided in the table for each TSS, which allows for customized filtering. The tables consist of the following columns:

*Pos*: The position of the TSS.
*Strand*: The strand of the TSS.
*stepHeight*: The expression height change at the position of the TSS. This relates to the number of reads starting at this position.
*stepFactor*: The factor of height change at the position of the TSS.
*enrichmentFactor*: The enrichment factor at the position of the TSS.
*classCount*: The number of classes to which this TSS was assigned.
*Locus_tag*: The locus tag of the gene to which the classification relates.
*Product*: The product description of this gene.
*UTRlength*: The length of the untranslated region between the TSS and the respective gene (nt). (Only applies to 'primary' and 'secondary' TSS.)
*GeneLength*: The length of the gene (nt).
*Primary*: Contains a '1' if the TSS was classified as 'primary' with respect to the gene stated in 'locusTag'.
*Secondary*: Contains a '1' if the TSS was classified as 'secondary' with respect to the gene stated in 'locusTag'.
*Internal*: Contains a '1' if the TSS was classified as 'internal' with respect to the gene stated in 'locusTag'.
*Antisense*: Contains a '1' if the TSS was classified as 'antisense' with respect to the gene stated in 'locusTag'.
*Automated*: Contains a '1' if the TSS was detected automatically.
*Manual*: Contains a '1' if the TSS was annotated manually.
*Putative sRNA*: Contains a '1' if the TSS might be related to a novel sRNA.
*Putative asRNA*: Contains a '1' if the TSS might be related to an asRNA.
*Sequence -50 nt upstream + TSS (51nt)*: Contains the base of the TSS and the 50 nucleotides upstream of the TSS.

## Supplementary References


1. Fouts DE, Mongodin EF, Mandrell RE, Miller WG, Rasko DA, et al. (2005) Major structural differences and novel potential virulence mechanisms from the genomes of multiple *Campylobacter* species. PLoS Biol 3: e15.
2. Camacho C, Coulouris G, Avagyan V, Ma N, Papadopoulos J, et al. (2009) BLAST+: architecture and applications. BMC Bioinformatics 10: 421.
3. Edgar RC (2004) MUSCLE: multiple sequence alignment with high accuracy and high throughput. Nucleic Acids Res 32: 1792-1797.
4. Talavera G, Castresana J (2007) Improvement of phylogenies after removing divergent and ambiguously aligned blocks from protein sequence alignments. Syst Biol 56: 564-577.
5. Guindon S, Dufayard JF, Lefort V, Anisimova M, Hordijk W, et al. (2010) New algorithms and methods to estimate maximum-likelihood phylogenies: assessing the performance of PhyML 3.0. Syst Biol 59: 307-321.
6. Le SQ, Gascuel O (2008) An improved general amino acid replacement matrix. Mol Biol Evol 25: 1307-1320.
7. Creevey CJ, McInerney JO (2005) Clann: investigating phylogenetic information through supertree analyses. Bioinformatics 21: 390-392.
8. Letunic I, Bork P (2011) Interactive Tree Of Life v2: online annotation and display of phylogenetic trees made easy. Nucleic Acids Res 39: W475-478.
9. Berezikov E, Thuemmler F, van Laake LW, Kondova I, Bontrop R, et al. (2006) Diversity of microRNAs in human and chimpanzee brain. Nat Genet 38: 1375-1377.
10. Hoffmann S, Otto C, Kurtz S, Sharma CM, Khaitovich P, et al. (2009) Fast mapping of short sequences with mismatches, insertions and deletions using index structures. PLoS Comput Biol 5: e1000502.
11. Nicol JW, Helt GA, Blanchard SG, Jr., Raja A, Loraine AE (2009) The Integrated Genome Browser: free software for distribution and exploration of genome-scale datasets. Bioinformatics 25: 2730-2731.
12. Sharma CM, Hoffmann S, Darfeuille F, Reignier J, Findeiss S, et al. (2010) The primary transcriptome of the major human pathogen Helicobacter pylori. Nature 464: 250-255.
13. Skouloubris S, Thiberge JM, Labigne A, De Reuse H (1998) The *Helicobacter pylori* UreI protein is not involved in urease activity but is essential for bacterial survival *in vivo*. Infect Immun 66: 4517-4521.
14. Stingl K, Brandt S, Uhlemann EM, Schmid R, Altendorf K, et al. (2007) Channel-mediated potassium uptake in *Helicobacter pylori* is essential for gastric colonization. EMBO J 26: 232-241.
15. Hansen CR, Khatiwara A, Ziprin R, Kwon YM (2007) Rapid construction of *Campylobacter jejuni* deletion mutants. Lett Appl Microbiol 45: 599-603.
16. Chaudhuri RR, Yu L, Kanji A, Perkins TT, Gardner PP, et al. (2011) Quantitative RNA-seq analysis of the transcriptome of *Campylobacter jejuni*. Microbiology 157: 2922-2932.
